# Profiling the role of m6A effectors in the regulation of pluripotent reprogramming

**DOI:** 10.1186/s40246-024-00597-6

**Published:** 2024-04-02

**Authors:** Wenjun Wang, Lei Zhou, Hui Li, Tingge Sun, Xue Wen, Wei Li, Miguel A. Esteban, Andrew R. Hoffman, Ji-Fan Hu, Jiuwei Cui

**Affiliations:** 1 Cancer Center, The First Hospital of Jilin University, Changchun, Jilin 130021 China; 2 VA Palo Alto Health Care System, Stanford University School of Medicine, Palo Alto, CA 94304 USA; 3 Guangzhou Institutes of Biomedicine and Health, Chinese Academy of Sciences, Guangzhou, Guangdong 510530 PR China

**Keywords:** m6A, Epigenetics, IGF2BPs, iPSC, Stem cells, Reprogramming

## Abstract

The N^6^-methyladenosine (m^6^A) RNA modification plays essential roles in multiple biological processes, including stem cell fate determination. To explore the role of the m6A modification in pluripotent reprogramming, we used RNA-seq to map m6A effectors in human iPSCs, fibroblasts, and H9 ESCs, as well as in mouse ESCs and fibroblasts. By integrating the human and mouse RNA-seq data, we found that 19 m6A effectors were significantly upregulated in reprogramming. Notably, IGF2BPs, particularly *IGF2BP1*, were among the most upregulated genes in pluripotent cells, while *YTHDF3* had high levels of expression in fibroblasts. Using quantitative PCR and Western blot, we validated the pluripotency-associated elevation of IGF2BPs. Knockdown of *IGF2BP1* induced the downregulation of stemness genes and exit from pluripotency. Proteome analysis of cells collected at both the beginning and terminal states of the reprogramming process revealed that the IGF2BP1 protein was positively correlated with stemness markers SOX2 and OCT4. The eCLIP-seq target analysis showed that IGF2BP1 interacted with the coding sequence (CDS) and 3’UTR regions of the *SOX2* transcripts, in agreement with the location of m6A modifications. This study identifies *IGF2BP1* as a vital pluripotency-associated m6A effector, providing new insight into the interplay between m6A epigenetic modifications and pluripotent reprogramming.

**Supplementary Information:**

The online version contains supplementary material available at 10.1186/s40246-024-00597-6.

## Introduction

Various cellular RNA modifications are important posttranscriptional regulators of gene function programs, and they can play a vital role in regulating the organized temporal dynamics and spatial patterning of gene expression [1–3]. Up to 150 distinct chemical RNA modifications have been identified so far. The development of next-generation sequencing and sensitive epigenetic analytic tools have enabled us to investigate and map modified RNA sites in various cells at high-resolution [4]. One of the best-studied modifications is N6-methyladenosine (m6A) [1, 5]. The m6A modification has been reported to regulate the physiology and metabolism (splicing, stability, exportation, and protein translation) of various types of RNA, including mature messenger RNA (mRNA) [5, 6], transfer RNA (tRNA) [7], ribosomal RNA (rRNA) [8], circular RNA (circRNA) [9, 10], and noncoding RNA (ncRNA) [7]. The diversity of m6A modified RNA species highlights the importance and complexity of m6A modification in multiple cells [1]. More importantly, multiple effectors involved in m6A pathways have been identified, including m6A methyltransferases as the “writers”, demethylases as the “erasers”, and m6A-binding proteins as the “readers”. They respectively install, remove, and recognize the m6A modification (reviewed by Shi et al.) [1].

Recent studies have shown that m6A plays a role in a variety of eukaryotic biological processes, including stem cell reprogramming [11–13], normal development [14], solid tumors [15, 16], hematologic malignancies [17], and other diseases [18]. Bastista et al. demonstrated that m6A methylomes played a role in controlling embryonic stem cell fate [13]. Zhang et al. reported that m6A was essential for the emergence of hematopoietic stem/progenitor cells (HSPCs) during embryogenesis in zebrafish [19].

Pluripotent stem cells (PSCs) are attractive and promising cell therapies for various injuries and diseases [20]. Two types of human PSCs have emerged as the pillars of stem cell therapy: embryonic stem cells (ESCs) and induced pluripotent stem cells (iPSCs) [21]. iPSCs, first reported by Yamanaka’s group in 2006, refer to the reprogramming of terminally differentiated cells into a pluripotent stage using a cocktail of stem cell transcription factors Oct4-Sox2-Klf4-c-Myc (OSKM) [20, 22, 23]. PSCs can be induced by somatic nuclei transfer [24] and small-molecule compounds [25, 26]. However, barriers in the reprogramming processes result in extremely low reprogramming efficiency, inhibiting the development of iPSCs for clinical use [27].

Cell reprogramming is dynamically regulated by a network of epigenetic signals. The epigenetic profile directly determines the developmental potential of stem cells, including pluripotency establishment, self-renewal, lineage-specific differentiation, as well as apoptosis, and stem cell aging. In this study, we aimed to assess the role of m6A RNA modifications in regulating stem cell fate by identifying m6A effectors that are associated with pluripotency. We have identified the insulin-like growth factor-2 mRNA-binding proteins(IGF2BP) family of genes, particularly *IGF2BP1*, as vital pluripotency-associated m6A effectors. Using eCLIP-seq and m6A-seq, we provide evidence for an important regulatory interplay between m6A epigenetic modifications and pluripotent reprogramming.

## Materials and methods

### Cell lines and cell culture

E14 mouse embryonic stem (ES) cells were purchased from ATCC and were maintained in the ESC medium containing KnockOut Dulbecco’s Modified Eagle Medium (DMEM, #10829018, Gibco), 10% bovine serum (FBS), l-glutamine (25030-081, Invitrogen), non-essential amino acids (#11140050, Gibco), penicillin/streptomycin (10378016, Gibco), and 2-mercaptoethanol and supplemented with Leukemia Inhibitory Factor (LIF, Sigma). Mouse embryonic fibroblasts (MEFs) were cultured from fetal mice and maintained in DMEM (#11965092, Gibco) containing 10% FBS, non-essential amino acids (#11140050, Gibco), and penicillin/streptomycin (10378016, Gibco). Human embryonic stem cell line (hESC) H9 and human iPSC line C11 [28] were cultured in mTeSR™1 media (#100–0276, STEMCELL Technologies) on tissue culture plates coated with Matrigel (#356234, BD Bioscience). Human fibroblast cell line SPF7 [29] was cultured in DMEM (#11965092, Gibco) containing 10% FBS, l-glutamine (25030-081, Invitrogen), non-essential amino acids (#11140050, Gibco), and penicillin/streptomycin (10378016, Gibco).

### RNA-seq to identify the expression of m6A effectors

Total RNA was extracted by TRIzol reagent (#15596026, Invitrogen) from human and mouse iPSCs and fibroblasts. The indexed libraries were prepared using Illumina’s TruSeq RNA Sample Prep Kit v2. Paired-end sequencing in triplicate was performed by Jilin Epigenomes Biotechnology (Jilin, PRC) using a HiSeq4000 (Illumina). After Seqtk filtering, clean reads for E14, mouse fibroblasts, H9, C11, and SPF7 were mapped to the human genome (genome version: GRCh38/hg38) and mouse genome (genome version: GRCm38.p4/mm10) for mRNAs and lncRNAs using the STAR software [30]. Gene counts were normalized to the values of Fragments Per Kilobase of transcript per Million mapped reads (FPKM). Cuffdiff was used to calculate the differentially expressed RNAs when the fold-change was > 2 and *P* < 0.05 with an unpaired two-sided t-test.

### RNA-seq data analysis

Differentially expressed genes were identified using the DESeq2 package in R language [31] with |log fold change (FC)| >1 and a false discovery rate (FDR) cutoff of 0.05 as the threshold value. The ggplot2, heatmap, and volcanoplot packages were used to visualize DEGs: red dots indicate up-regulation and blue dots indicate down-regulation in the volcano plot; different colors in the heatmap represent the trend of m6A effector expression in different cell lines.

We also collected and summarized the RNA-seq data of GSM1706720, which covers six human samples, including two fibroblast cell lines, two ESC lines (H9 and H7), and two hiPSCs [32]. The ggplot2 and heatmap were used to visualize DEGs.

### Real-time RT-PCR (qPCR)

Total RNA was isolated from cells using TRIzol (#15596026, Invitrogen) and then stored at − 80 °C. The cDNA generation was performed with a Bio-Rad Thermol Cycler using PrimeScript™ RT Master Mix (#RR036B, TAKARA). The qPCR was performed using 2x Sybr qPCR Super Mix (#TBS4001R-10, Tribo science) as previously described. The target amplification was performed by RT-PCR of 1 cycle at 95 °C for 2 min; 40 cycles at 95 °C for 15s, and 60 °C for 30s; and dissociation stage. The threshold cycle (Ct) values of target genes were assessed by quantitative PCR in triplicate using a sequence detector (ABI Prism 7900HT; Applied Biosystems) and were normalized over the Ct of the β-actin as control. The primers used in our study were listed in Additional file 1: Table S1.

### Western blot analysis

Whole-cell proteins were isolated from human and mouse stem cells and fibroblasts using RIPA lysis buffer (10% SDS, 1 mM DTT, and glycerin) and 3X Blue Loading Buffer (#7722S, CST). Protein samples were incubated with the following primary antibodies in 5% bovine serum albumin (BSA): anti-IGF2BP1 (1:1000 dilution, #ab184305, ABCAM), anti-IGF2BP2 (1:1000 dilution, #ab128175, ABCAM), anti-IGF2BP3 (1:1000 dilution, #ab177477, ABCAM), anti-YTHDF3 (#ab220161, ABCAM), anti-RBM15B (#ab300467, ABCAM), anti-SOX2 (1:1000 dilution, #ab97959, ABCAM), anti-OCT4 (1:1000 dilution, #ab19857, ABCAM), anti-NANOG (1:1000 dilution, #ab109250, ABCAM), and anti-β-actin (1:1000 dilution, #66009-1-Ig, Proteintech). Secondary antibodies were HRP-linked goat anti-mouse (1:1000 dilution, #ab6789, ABCAM), goat anti-rabbit (1:1000 dilution, #ab205718, ABCAM). Protein expression was assessed by NcmECL Ultra (#P10300, NCMbiotech) and detected on ChemiDoc MP Imaging System (#12003154, Bio-Rad). (Table 1)

**Table 1:** antibody list for western blot analysis

Name	Catalogue No.	Type	Company
anti-IGF2BP1	ab184305	Rabbit monoclonal [EPR18791]	ABCAM
anti-IGF2BP2	ab128175	Mouse monoclonal [1E3.01E5]	ABCAM
anti-IGF2BP3	ab177477	Rabbit monoclonal [EPR12021]	ABCAM
anti-YTHDF3	ab220161	Rabbit monoclonal [EPR21912-3]	ABCAM
anti-RBM15B	ab300467	Rabbit monoclonal [EPR25177-134]	ABCAM
anti-SOX2	ab97959	Rabbit polyclonal	ABCAM
anti-OCT4	ab19857	Rabbit polyclonal	ABCAM
anti-NANOG	ab109250	Rabbit monoclonal [EPR2027(2)]	ABCAM
anti-β-actin	66009-1-Ig	Mouse IgG2b	Proteintech
HRP-linked goat anti-mouse	ab6789	Goat Anti-Mouse IgG H&L (HRP)	ABCAM
HRP-linked goat anti-rabbit	ab205718	Goat Anti-Rabbit IgG H&L (HRP)	ABCAM

### Knockdown of IGF2BPs by siRNA

IGF2BPs siRNA were purchased from GenePharma (Shanghai GenePharma Co., Ltd, China). The siRNAs specific for IGF2BP1 mRNA (IGF2BP1-1, 5$\prime$-GCUCCCUAUAGCUCCUUUATT-3$\prime$; IGF2BP1-2, 5$\prime$-GGGAAGAGCUGGAGGCCUA-3$\prime$; IGF2BP1-3, 5$\prime$-UGAAUGGCCACCAGUUGGA-3$\prime$), IGF2BP2 mRNA (IGF2BP2-1, 5$\prime$-AGAAGUGAAGCUGGAAGCG-3$\prime$; IGF2BP2-2, 5$\prime$-GCUGAUAGUUGGAGCAUUU-3$\prime$; IGF2BP2-3, 5$\prime$-GGGAAGAUGUUAAGAUAUG-3$\prime$), and IGF2BP3 mRNA (IGF2BP3-1, 5$\prime$-AUGUAACCUAUUCCAGUAA-3$\prime$; IGF2BP3-2, 5$\prime$-UAAGGAAGCUCAAGAUAUA-3$\prime$; IGF2BP3-1, 5$\prime$-GAGCAAGACACAGACACUA-3$\prime$), and the scrambled siRNA control is 5$\prime$-UUCUCCGAACGUGUCACGUTT-3$\prime$. H9 and C11 cell lines were transfected with siRNAs using jetPRIME transfection reagent (#19Y0301L14, Polyplus transduction) according to the manufacturer. 24 h after transfection, cells were incubated with the fresh complete medium for another 48 h before protein and mRNA analysis.

### Lentivirus production and infection

Lentiviral inducible shIGF2BP1 cells were obtained by transduction of pLKO.1 puro (plasmid #8453, Addgene), containing either the shRNA or the shNC. The sense strands of shIGF2BP1-1 and shIGF2BP1-2 that we used are 5$\prime$-CCGGTGAAGATCCTGGCCCATAATACTCGAGTATTATGGGCCAGGATCTTCATTTTTG − 3$\prime$ and 5’- CCGGGCAGTGGTGAATGTCACCTATCTCGAGATAGGTGACATTCACCACTGCTTTTTG − 3’. All the constructs were verified by sequencing. All transfection experiments were performed using Lipofectamine 2000 (#11668019, Thermo Fisher) and the production of lentivirus supernatant was described previously. The efficiency of gene silencing was evaluated at 24- and 48-hours post-transfection by real-time qPCR, and at 72 h by Western blot analysis.

### IF of stem cell markers

IF was used to examine the expression and location of the stem gene proteins (SOX2 and OCT4) in stem cells. Briefly, cells were fixed by freshly made 4% paraformaldehyde for 10 min at room temperature, permeabilized with freshly made 0.5% v/v Triton X-100/PBS on ice for 5 min, then blocked in 1% w/v BSA for 30 min at room temperature. After incubation with primary antibodies diluted in 1% BSA overnight at 4 °C, samples were washed three times in PBST for 5 min each. The following antibodies were used in the immunostaining: rabbit anti-SOX2 (1:100 dilution, #ab97959, ABCAM), anti-OCT4 (1:100 dilution, ab19857, ABCAM). The cell samples were subsequently incubated with Alexa Fluor 647-labeled goat anti-rabbit IgG(H + L) secondary antibodies (1:500, #A0468, Beyotime) for 1 h at room temperature. After washing three times with PBS, samples were counterstained with DAPI (D1306, Invitrogen). Fluorescence images were acquired with a confocal laser scanning microscope (#FV3000, Olympus).

### eCLIP data analysis

We collected the IGF2BPs eCLIP dataset GSE78509 based on GPL11154 platform (Illumina HiSeq 2000) [33]. The protein-RNA complexes in H9 cells were immunoprecipitated using IGF2BP1-3 antibodies and rabbit IgG [33]. Besides, a parallel Size-Matched Input (SMInput) library was generated as the control without any anti-RNA binding protein (anti-RBP) antibodies [33]. The raw data were downloaded from GEO and then processed in R language. eCLIP-seq was analyzed by gene enrichment analysis for GO (http://geneontology.org) and KEGG (https://www.genome.jp/kegg/) using DAVID v6.8 (https://david.ncifcrf.gov/). Enriched pathways were identified according to FDR ≤ 0.05.

### m6A-IP data analysis

To analyze the m6A methylome of stem genes RNA in stem cells, we collected the m6A-IP dataset GSE54365 based on GPL16791 platform (Illumina HiSeq 2500). We selected a dynamic system wherein human fibroblasts undergoing reprogramming into iPSC following doxycycline-induced expression of polycistronic OCT4-KLF4-MYC-SOX2, and hESCs were used for further analysis. Eight samples were involved including GSM1339395 human_hESC, GSM1339396 human_hESC_input, GSM1339407 human_OKMSiPSC (fibroblasts fully reprogrammed into iPSC), GSM1339408 human_OKMSiPSC_input, GSM1339403 human_OKMSfibroplusDox (5 days post-induction with Dox), GSM1339404, human_OKMSfibroplusDox_input, GSM1339405 human_OKMSfibrominusDox (5 days after not inducing with Dox), GSM1339406 human_OKMSfibrominusDox_input [34]. Integrative Genomics Viewer (IGV) was used to analyze the m6A modification of stem genes (including SOX2, OCT4, NANOG, and KLF4).

### Quantitative mass spectrometry data anlaysis

We collected quantitative mass spectrometry data from five samples IMR90_Fibro (IMR90 fetal fibroblasts), 4Skin_Fibro (foreskin fibroblasts), IMR90_iPS (human iPSCs through the reprogramming of IMR90 fetal fibroblasts), 4Skin_iPS (human iPSCs through the reprogramming of foreskin fibroblasts), and hESCs (HES-3) [35]. After calculating the hESCs/hiPSCs and Fibroblasts/hiPSCs, the heatmap and ggplot2 packages were used to visualize DEGs. Correlation analysis was conducted between m6A effectors and stem genes (including SOX2 and OCT4).

### IGF2BP1 cross-linking immunoprecipitation (CLIP)

UV-crosslinked C11 cells were lysed in CLIP lysis buffer and sonicated. Anti-IGF2BP1 (#8482, CST) and rabbit IgG (#2729, CST) were used to immunoprecipitated IGF2BP1-RNA complexes, respectively. Meanwhile, an aliquot was saved as the input without anti-RBP antibodies. After reverse crosslinking, RNAs were purified and reverse transcripted. Enrichment of the IGF2BP1-binding cDNAs was quantitated by qPCR. The specific gene primers were listed in Table S3.

### m6A-RNA immunoprecipitation (RIP)

C11 cell line was collected and lysed by lysis buffer for RIP. The cell extract was incubeated with magnetic beads cojugated with anti-m6A antibodies (#ab208577, ABCAM) or mouse lgG (#SC-2025, Santa cruz Biotechnology), respectively. The m6A modified RNA was immunoprecipitated after protein digestion by proteinase K. After purification and reverse transcription, enrichment of the m6A modified cDNAs was quantitated by qPCR using target gene primers (Table S3).

### Statistical analysis

All numerical data are presented as mean ± standard deviation of triplicate assays. The statistical significances were determined using Student’s two-tail t-test, where *p* < 0.05 was considered statistically significant. In all figures, the statistical significances were indicated with * if *P* < 0.05 or ** if *P* < 0.01.

## Results

### Identification of IGF2BP1 as a pluripotency-associated m6A effector

**Fig. 1:**
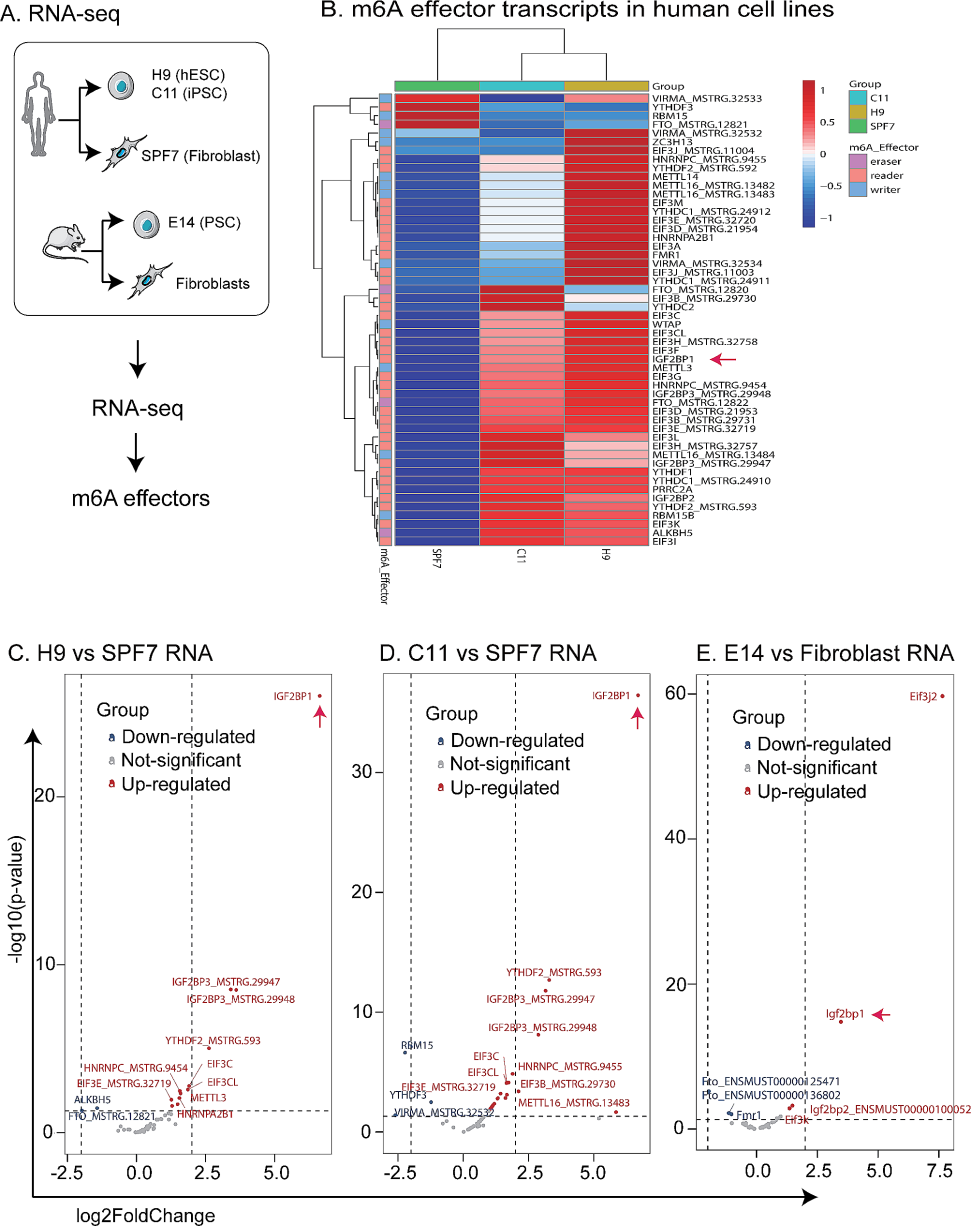
Identification of IGF2BP1 as an important pluripotency-associated m6A effector. **A**. Schematic diagram of the RNA-seq using human and mouse pluripotent stem cells and fibroblast. C11 iPSC: induced pluripotent stem cell line C11; H9 hESC: human embryonic stem cell line H9 (positive control); PSC: Pluripotent stem cells; SPF7: human skin fibroblasts. **B** Heatmaps depicting expression levels of m6A effector transcripts in human pluripotent stem cells (H9 and C11) and fibroblasts (SPF7). The expression levels were normalized by the row. **C-E**. Volcano plots of genes downregulated/upregulated in the indicated pairwise comparisons. Only genes with an average read count of three or greater across all samples are included. Genes with a log2-fold-change > 1or < − 1, and False Discovery Rate (FDR) < 0.05 are shown in red or blue (*n* = 1 for each cell line). Genes without significant difference (|log2-fold-change|<1 or FDR > 0.05) are shown in grey

To delineate the role of m6A modifications in pluripotency, we used RNA transcriptome sequencing (RNA-seq) to profile the m6A effectors that are associated with pluripotent reprogramming (Fig. 1A). We screened all the current m6A effectors that have been reported [1]. RNA-seq was performed for initial screening of m6A effectors that are differentially expressed between human iPSCs (C11) and unreprogrammed human control fibroblasts (SPF7). ESCs (H19) were used as the positive control. For comparison, RNA-seq was also performed in mouse E14 ESCs and fibroblast control cells.

The differential analysis revealed that most of the m6A effectors were upregulated in human PSCs as compared with fibroblast controls (Fig. 1B, S1). Notably, the IGF2BP family (IGF2BP1, IGF2BP2, IGF2BP3), especially IGF2BP1, were the most significantly upregulated m6A effectors in C11 iPSCs and H9 ESCs (Fig. 1C and D). Similarly, IGF2BP1 was also the second most highly upregulated gene in mouse E14 ESCs as compared with mouse fibroblasts (Fig. 1E).

To identify the pluripotency-associated m6A effectors, we integrated the human stem cells’ RNA-seq data with the mouse RNA-Seq data (Figs. S2A-S2D). By combining these datasets, we identified 33 common upregulated and 2 downregulated m6A effectors in two human stem cell lines (H9 and C11, Fig. S2A). Moreover, using a Venn diagram analysis, we found 19 m6A effectors that were significantly enriched in both human and mouse stem cells. Again, IGF2BP1 was one of the most highly upregulated genes (Figs. S2B-S2D). We have also reviewed the RNA-seq dataset of GSM1706720, which covers six human samples, including two fibroblast cell lines, two ESC lines (H9 and H7), two hiPSCs [32]. Again, m6A effectors, particularly the IGF2BPs family, were upregulated in ESCs and hiPSCs (Fig. S1C). In summary, the elevated expression of the IGF2BP family, particularly IGF2BP1, in pluripotent stem cells suggests a critical role for these pluripotency-associated m6A effectors in stem cell fate determination.

### IGF2BP1 is associated with the pluripotent stages

**Fig. 2:**
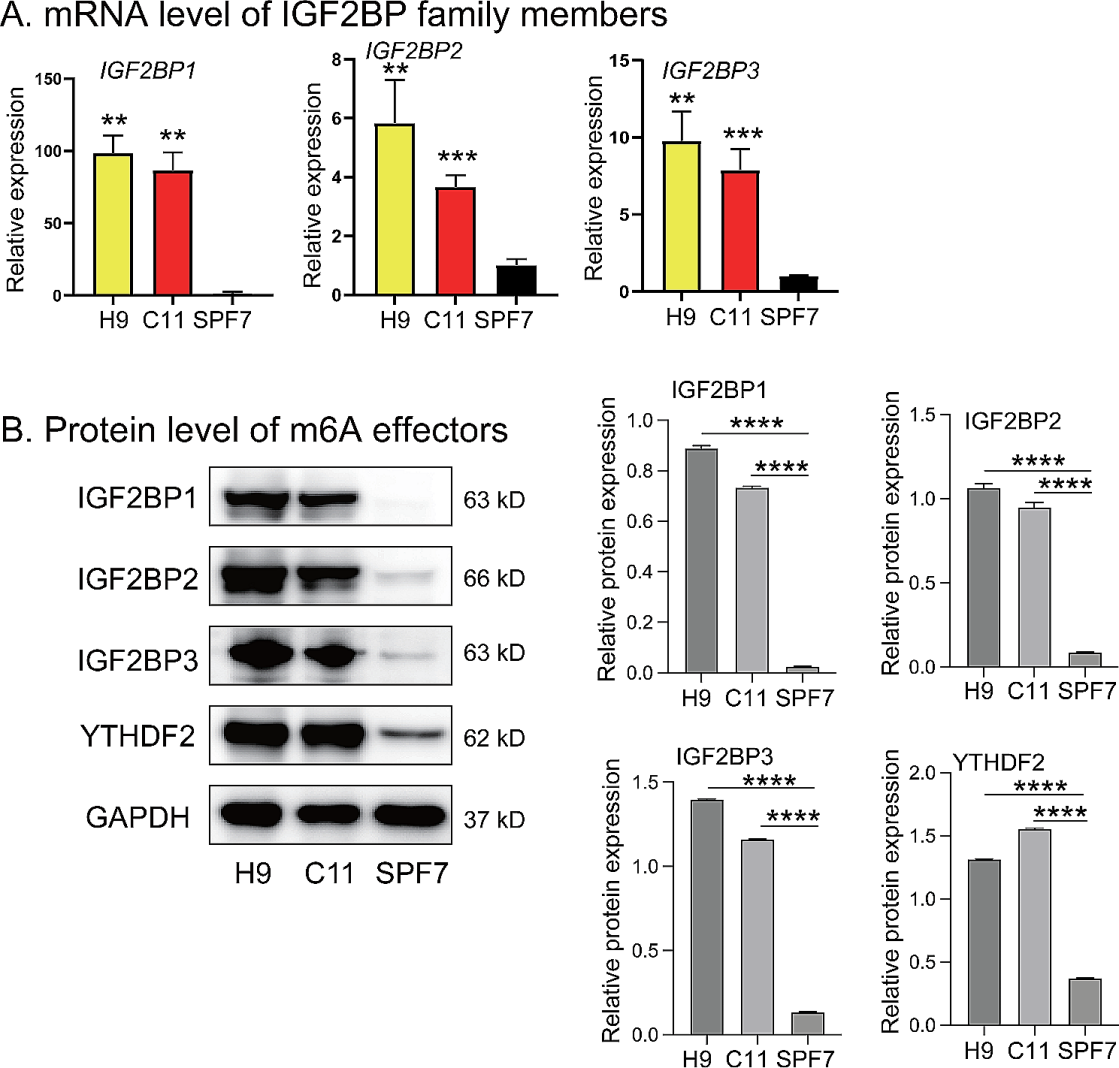
Differential expression of the IGF2BP gene family in stem cells. **A**. Quantitation of mRNA abundance of IGF2BP family members. Human pluripotent stem cells (H9 and C11) and fibroblasts (SPF7) were collected and the expression of IGF2BP family members was evaluated by real-time RT-PCR. The expression levels were normalized using β-Actin. The data are presented as the mean ± SD, and three independent experiments were performed. * *p* < 0.05; ** *p* < 0.01; *** *p* < 0.001; **** *p* < 0.0001 (t-test). **B**. Left panel, protein level of m6A effectors by Western blot. Right panel, quantification of the Western blot image using ImageJ software

We next performed quantitative real-time PCR (qPCR) to measure the expression of the IGF2BP family of genes in stem cells and in control fibroblasts. In agreement with the RNA-seq data, we found that the expression levels of all the IGF2BP family members were closely associated with the status of pluripotency (Fig. 2A). In addition, we also found greater abundance of YTHDF2 (YTH N6-methyladenosine RNA binding protein 2), METTL3 (methyltransferase-like 3), and RBM15B (RNA binding motif protein 15B), but decreased abundance of YTHDF3 in pluripotent stem cells (Fig. S3).

Western blot analysis was performed to validate the above findings. We observed relatively high protein abundance of IGF2BPs and YTHDF2 in the C11 and H9 pluripotent stem cells, but very low protein levels in the fibroblast controls (Fig. 2B). The IGF2BPs Western blot results indicate the existence of doublet bands, especially with prolonged exposure time, which implies that pluripotent stem cells may express varied isoforms of IGF2BPs.

### Knockdown of IGF2BP1 downregulates stemness gene expression

**Fig. 3:**
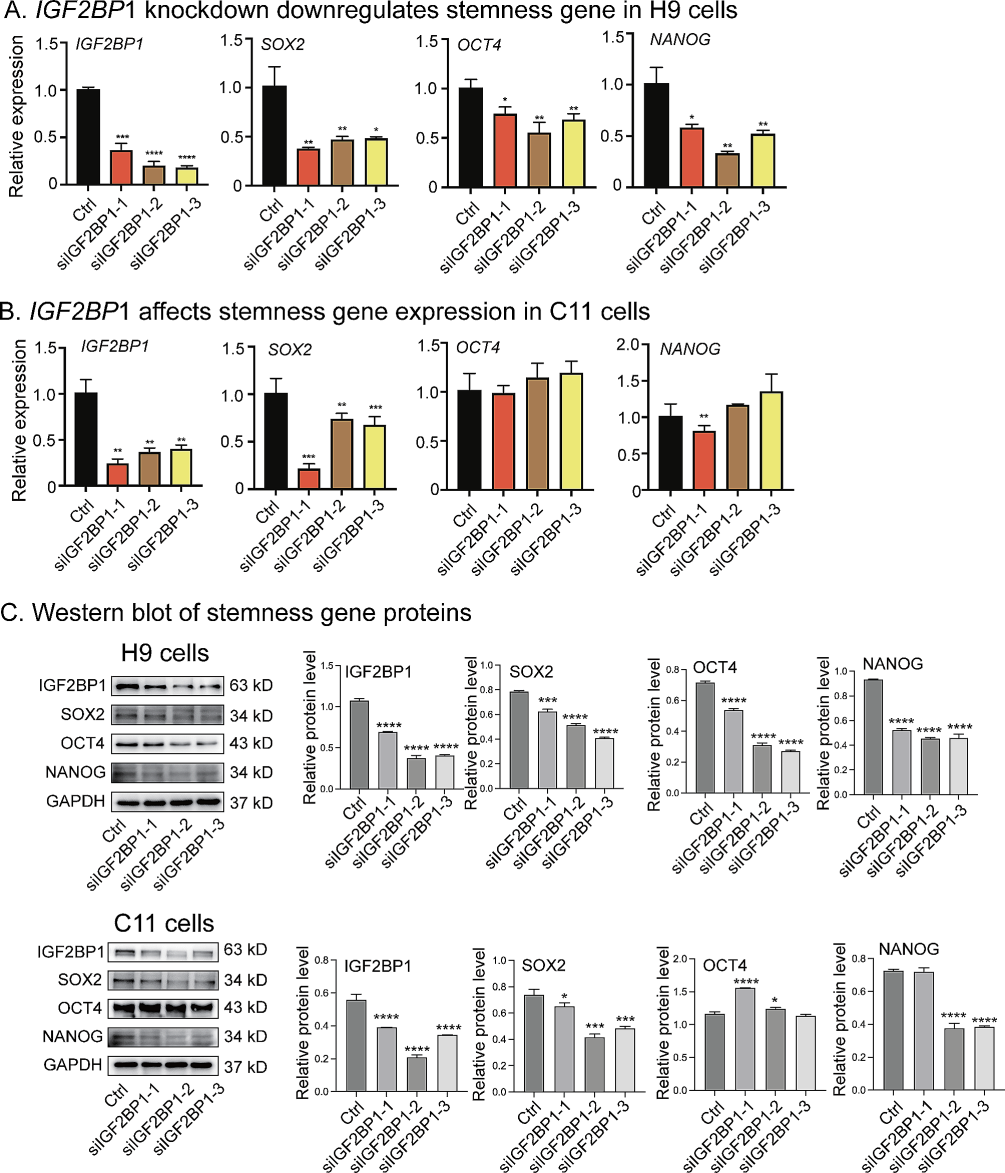
Knockdown of IGF2BP1 downregulates Stemness genes. **A-B**. Quantitation of stemness genes by real-time PCR in H9 and C11 cells. Cells were collected 48 h after three siIGF2BP1 treatments. β-Actin was used as the control. The decreased expression of *IGF2BP1* and *SOX2* transcripts was observed after transduction with IGF2BP1 siRNA in H9 and C11 cells. **C**. Left panel, Western blot of stemness genes. Cells were collected for Western blotting 72 h after siIGF2BP1 treatment in H9 and C11 cells. Right panel, quantification of the Western blot image using ImageJ software

To examine the role of IGF2BPs in stem cells, we used three siRNAs to knock down IGF2BP1 in H9 cells (Fig. 3A, panel 1) and C11 cells (Fig. 3B, panel 1). Knockdown of IGF2BP1 led to a significant reduction in the expression of the three stemness genes *OCT4*, *SOX2*, and *NANOG* in H9 cells (Fig. 3A, panels 2–4). In C11 cells, however, the expression of *OCT4* and *NANOG* was less influenced by IGF2BP1 knockdown. Similar data were also obtained in the Western blot assay (Fig. 3C). Upon immunodetection of NANOG, non-specific bands were observed. This is likely due to the non-specificity of the primary antibody acquired from ABCAM.

Knockdown of other IGF2BP family proteins IGF2BP2 and IGF2BP3, also affects the expression of these stemness genes to variable extents (Figs. S4-S5).

### IGF2BP1 knockdown affects pluripotency

**Fig. 4:**
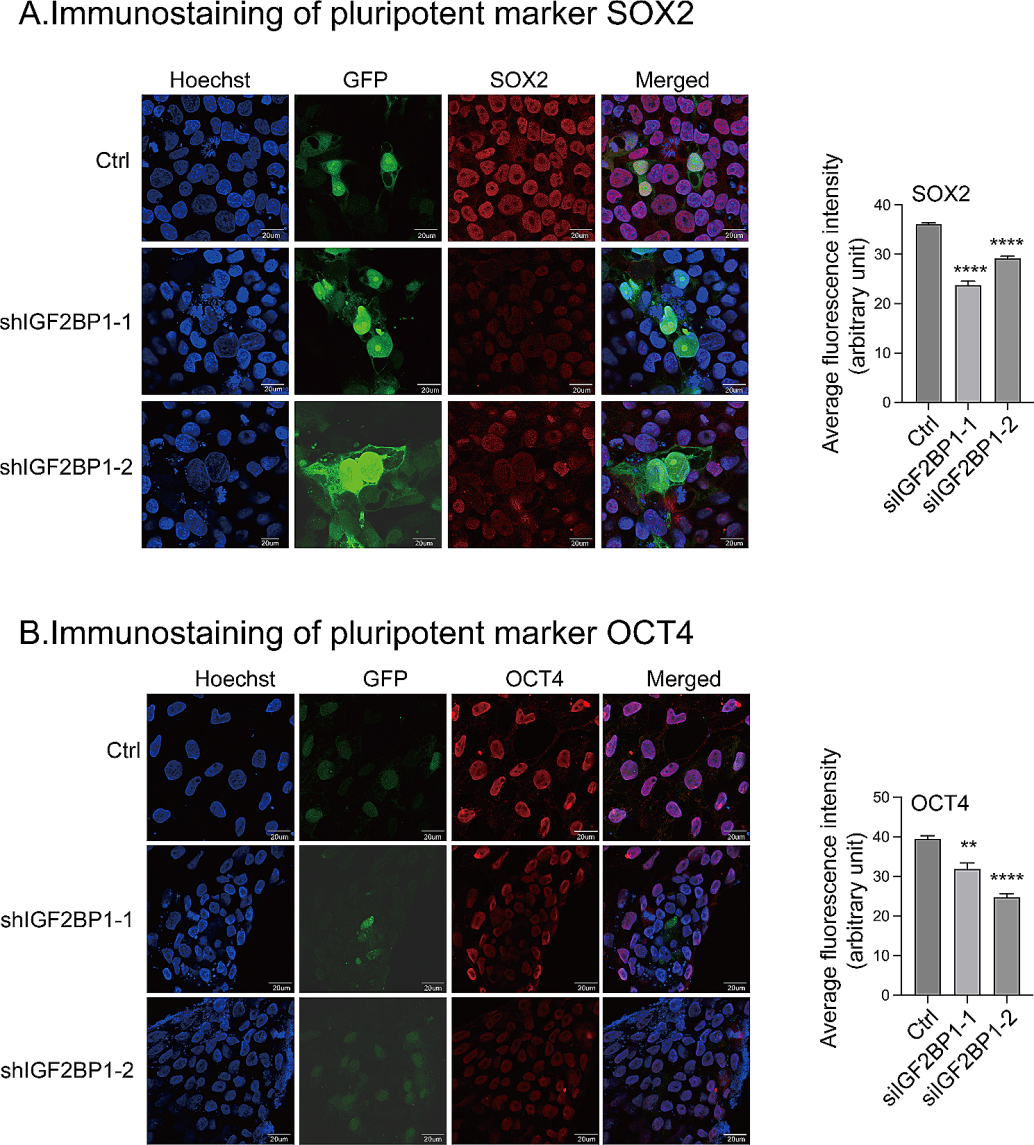
IGF2BP1 knockdown altered pluripotency in H9 cells. H9 cells were transduced using two shRNAs (shIGF2BP1-2, eGFP, green). Pluripotency was assessed by immunostaining of markers SOX2 (**A** left, panel 3, red) and OCT4 (**B** left, panel 3, red) at 72-hour post-transduction. Scale bar, 20 μm. Quantification of the intensity of SOX2 (**A**, right panel) and OCT4 (**B**, right panel) fluorescence is represented as mean ± SEM (arbitrary units, *N* = 3)

In addition to siRNAs, we also used short hairpin RNAs (shrines)-mediated gene editing to target IGF2BP1 and generated two IGF2BP1 knockdown H9 lines. Pluripotency was examined by immunofluorescence staining (IF) of stemness protein markers SOX2 and OCT4. After IGF2BP1 knockdown, we found a significant decrease in the expression of stemness markers SOX2 and OCT4. These results suggest that the knockdown of IGF2BP1 alters pluripotency in H9 cells (Fig. 4A-B).

### Proteome analysis reveals an association of IGF2BP1 with stemness markers SOX2 and OCT4 in reprogramming

To further delineate the role of IGF2BPs, we examined the association of m6A effector protein expression with pluripotent reprogramming using five published online datasets reported by Munoz et al. [35], including IMR90_Fibro (IMR90 fetal fibroblasts), 4Skin_Fibro (foreskin fibroblasts), IMR90_iPS (human iPSCs through the reprogramming of IMR90 fetal fibroblasts), 4Skin_iPS (human iPSCs through the reprogramming of foreskin fibroblasts), and the positive control hESCs (HES-3). These cells were collected at different stages of reprogramming. The data from two iPSCs and their parental fibroblast cell lines enabled us to compare the proteome at both the beginning and the end of the reprogramming process.

**Fig. 5:**
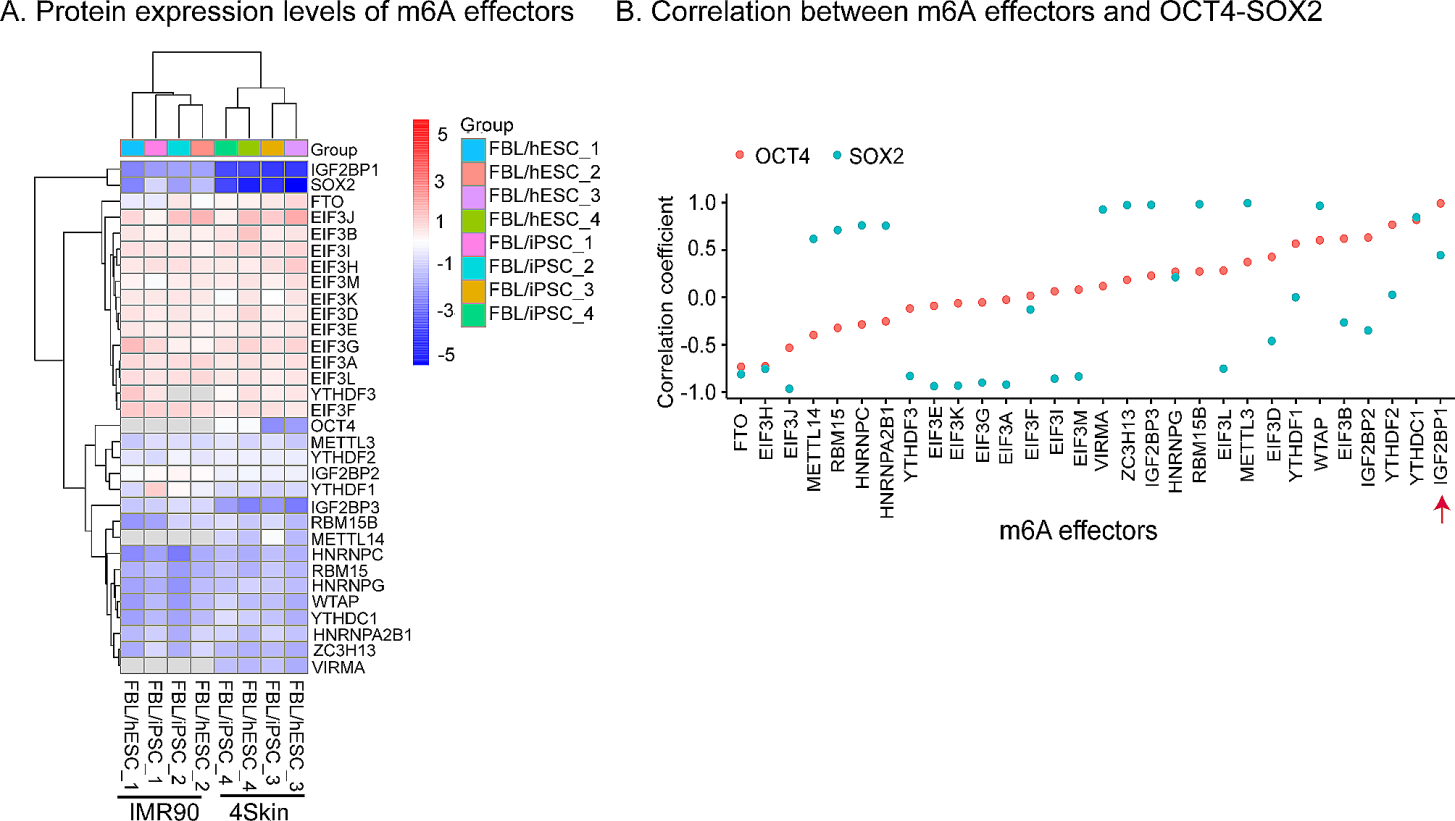
IGF2BP1 protein abundance is correlated with stemness markers OCT4 and SOX2. **A**. Heatmaps depicting protein expression levels of m6A effectors in Hess/hiPSCs and Fibroblasts/hiPSCs from five samples, including IMR90_Fibro (IMR90 fetal fibroblasts), 4Skin_Fibro (foreskin fibroblasts), IMR90_iPS (human iPSCs through the reprogramming of IMR90 fetal fibroblasts), 4Skin_iPS (human iPSCs through the reprogramming of foreskin fibroblasts), and Hess (HES-3). **B**. Correlations (Pearson correlation coefficient, Y-axis) between different m6A effectors (X-axis) and OCT4 and SOX2

Reanalysis of these proteomic data showed that most of the m6A effector proteins were differentially expressed between the parental fibroblasts and iPSCs (Fig. 5A). IGF2BP family proteins were enriched in stem cells, while the eukaryotic translation initiation factors (EIFs) family displayed very high expression in the fibroblasts. These results further indicate that IGF2BP family proteins are highly expressed in cells that have undergone complete reprogramming.

We next tested if the protein levels of m6A effectors were correlated with the levels of stem gene proteins using the same datasets by Munoz et al. [35]. There was a positive correlation between the expression level of IGF2BP family members, YTHDF (YT521-B homology Domain-containing Family) family members, YTHDC1 (YTH Domain Containing 1), and the stemness genes, SOX2 and OCT4 (Fig. 4B). IGF2BP1 protein had the highest correlation coefficient with stemness marker OCT4 and a modest correlation coefficient with SOX2. The correlation between the abundance of IGF2BP1 protein and stemness markers SOX2 and OCT4 suggests an important role for this m6A effector in regulating pluripotent reprogramming.

### Enhanced UV crosslinking and immunoprecipitation (eCLIP) identification of IGF2BP target genes

To delineate the mechanisms underlying the role of IGF2BPs, we used the eCLIP library data published by Conway et al. [33] to examine the interaction of IGF2BPs with their targets in stem cells. IGF2BP 1–3 proteins exhibited significant binding preferences to coding exons (CDS) and 3$\prime$ untranslated regions (3$\prime$UTRs) of mature mRNAs (Figs. S6A-S6B).

We utilized these IGF2BP1 eCLIP data to conduct Gene Ontology (GO) and Kyoto Encyclopedia of Genes and Genomes (KEGG) analyses (Figs. S6C-S6D). The target genes of IGF2BP1 were involved with ribonucleoprotein complex biogenesis, RNA catabolic process, mRNA catabolic process, RNA splicing, and mRNA processing pathways. In the cellular component, the target genes of IGF2BP1 were enriched in the ribosome and ribosomal subunit pathways. In the KEGG analysis, the target genes of IGF2BP1 were found to be involved in protein processing in the endoplasmic reticulum, RNA transport, and spliceosome pathways. The spliceosome can be involved in pre-mRNA splicing, whereby introns are excised from pre-mRNA to generate mature mRNA [36]. These results suggest that IGF2BP1 can be involved in the modification and translation of various RNAs in stem cells.

**Fig. 6:**
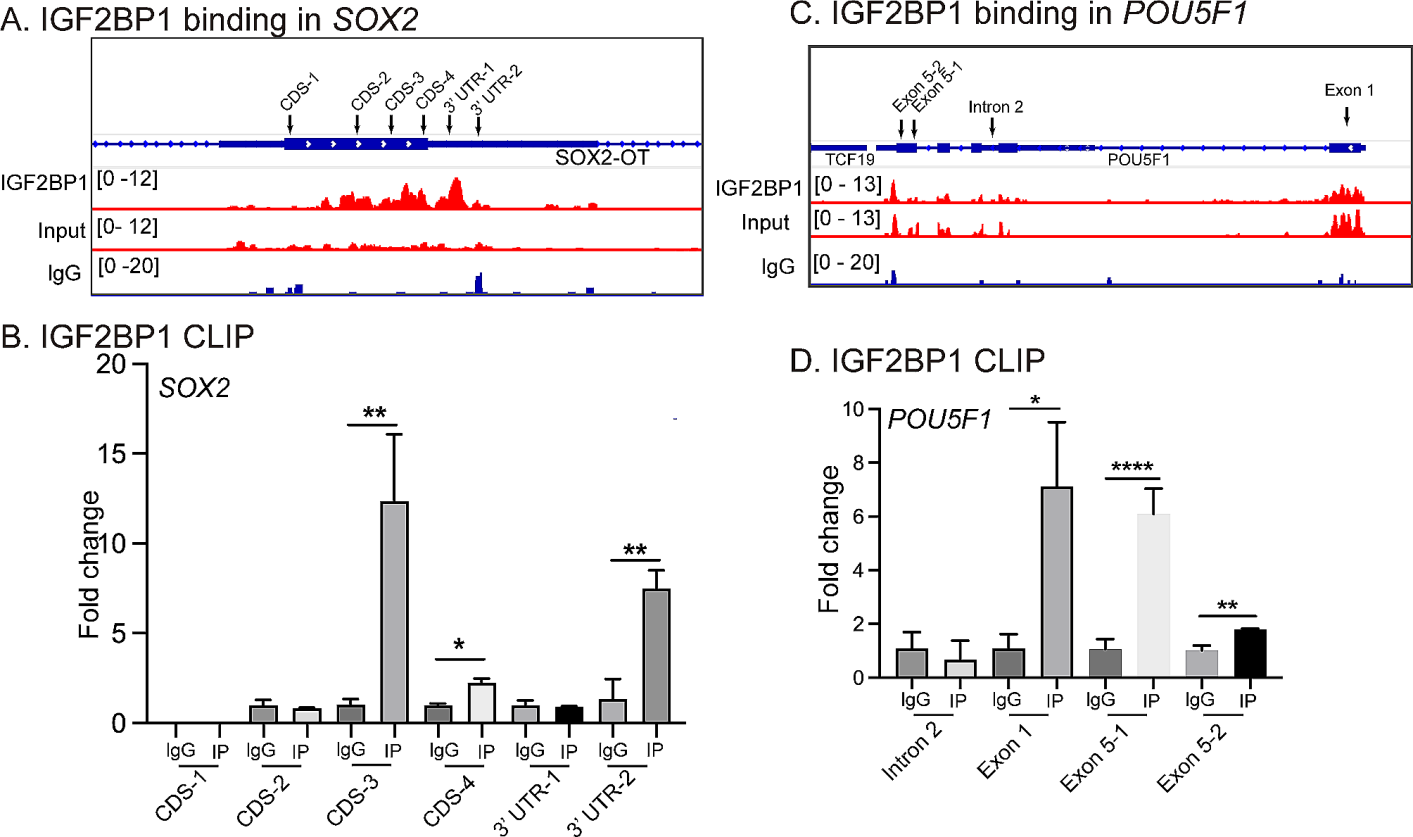
IGF2BP1 binds to stem genes in pluripotent stem cells. **A** and **C**. IGF2BP1-binding sites and eCLIP signals in H9 cell at the *SOX2* (**A**) and *POU5F1* (*OCT4*, **C**) loci. **B **and **D** CLIP fold-enrichment of IGF2BP1 binding in the *SOX2* (**B**), *POU5F1* (**D**) loci in C11 cells. IgG: CLIP control group, IP: IGF2BP1 CLIP group

We then determined whether the m6A reader IGF2BP1 bound to and regulated stemness genes. Through eCLIP target analysis, we found that IGF2BP1 interacted primarily with *SOX2* in H9 pluripotent stem cells (Fig. 6A, S7A). IGF2BP1 was enriched in the CDS and 3’UTR of *SOX2* in H9 cells. S100A4 (fibroblast-specific protein 1, FSP1), a collagen type VI alpha 2 chain (COL6A2) that is absent in stem cells, was used as the negative control. S100A4 is highly expressed in fibroblasts and is recognized as a fibroblast marker [37]. COL6A2 encodes the α2(VI) chain of the extracellular matrix (ECM) protein collagen type VI [38]. The fold-enrichment score of IGF2BP1 binding to *SOX2* was higher than the binding to S100A4 and COL6A2 (Fig. S7B). However, the fold-enrichment score of IGF2BP1 binding to *OCT4* (*POU5F1*) was not significantly elevated compared with the control (Fig. S7C-S7D).

Finally, we performed the cross-linking immunoprecipitation (CLIP) assay in C11 cells. The CLIP data also demonstrated the binding of IGF2BP1 to both the *SOX*2 (Fig. 6B) and *OCT4* (Fig. 6D) loci.

### m6A modification of four stem genes in stem cells by m6A-IP

**Fig. 7:**
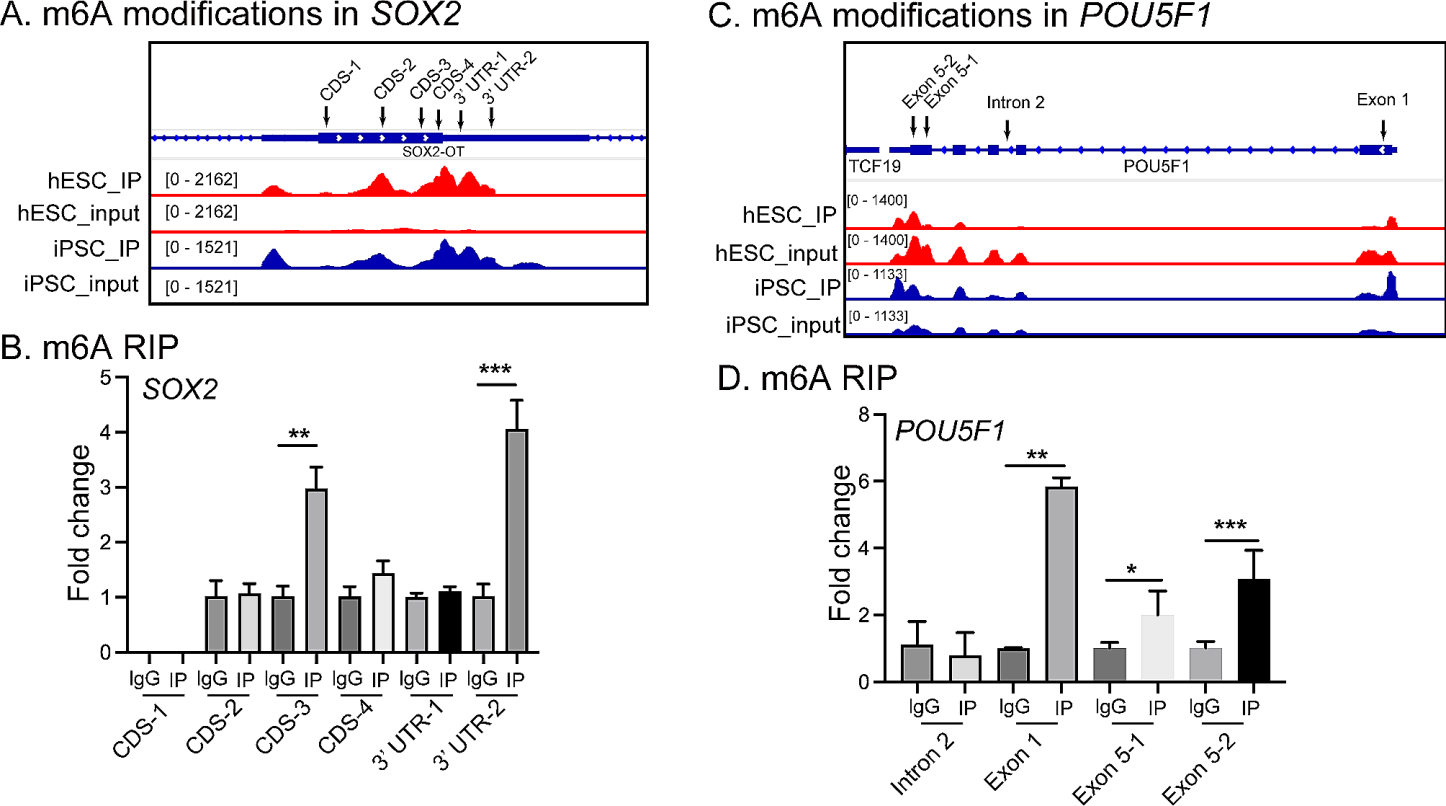
Reprogramming-associated m6A modifications in ***SOX2*** and ***OCT4*** gene transcripts. **A** and **C**. m6A signal in stem gene transcripts involved in stem cell reprogramming. m6A-IP enrichment signals were the read depth of m6A immunoprecipitation data in human hESC (red), iPSC (fibroblasts fully reprogrammed into iPSC, blue). Transcript regions are depicted as coding exons which are represented by blocks connected by horizontal lines representing introns. **B** and **D**. m6A RIP in *SOX2* (**B**) and *OCT4* (*POU5F1*, **D**). IgG: m6A RIP control group, IP: m6A RIP group

We then used the m6A-immunoprecipitation (m6A-IP) dataset reported by Schwartz et al [34] to analyze the m6A modification in mRNAs of stem cell core pluripotency factors *SOX2, POU5F1* (*OCT4*), and *NANOG*. The analysis of the m6A-IP data showed that the mRNAs encoding core pluripotency regulators in stem cells, including *SOX2* and *POU5F1*, contained m6A-modified RNAs (Fig. 7A and C). In agreement with the IGF2BP1 binding, the 5’ and 3’ UTR regions of these stem genes had significantly elevated levels of m6A modification.

We also conducted the m6A-RNA immunoprecipitation (RIP) assay in C11 cells. Our m6A RIP data further verified a significant m6A modification in the CDS and UTR of *SOX2* and *OCT4*, respectively (Fig. 7B and D).

It was interesting to note that doxycycline-induced expression of a polycistronic *OCT4-KLF4-MYC-SOX2* also resulted in significantly upregulated m6A methylation levels of *SOX2, POU5F1, NANOG*, and *KLF4* mRNAs in human fibroblasts, emphasizing the role of m6A in reprogramming into iPSCs (Fig. S8). However, the 5’ and 3’ UTR regions of these four genes were not m6A-modified during stem cell reprogramming. The m6A modification of stem gene RNAs is mainly located in CDS regions (Fig. 7A and C). Thus, in stem cells, m6A targets include the ESC core pluripotency network and transcripts with dynamically controlled abundance during stem cell reprogramming.

## Discussion

Using RNA-seq, quantitative mass spectrometry data, and eCLIP-seq, we have identified *IGF2BP1* as a critical m6A effector that is involved in maintaining the pluripotent status of stem cells, including iPSCs and ESCs. These results demonstrate that m6A marks a subset of stemness genes in stem cells and plays a critical role in stem cell reprogramming. IGF2BP1 expression is increased in pluripotent stem cells where it binds to crucial pluripotency factor target mRNAs, especially *SOX2*.

IGF2BP 1–3, also known as IMP 1–3, are part of a family of zipcode-binding proteins that have six putative RNA-binding domains, i.e., two RNA recognition motifs and four human heterogeneous nuclear ribonucleoprotein K-homology domains [39, 40]. IGF2BPs are oncofetal proteins that are normally expressed at high levels during mammalian embryogenesis and development [40]. Members of the IGF2BP family, especially IGF2BP1, are highly expressed in stem cells of both mouse and human, demonstrating the conservation of IGF2BP physiology in mammalian ESCs. The IGF2BP family takes part in various RNA processing steps, e.g., RNA trafficking and localization [41], decay [42], translation, and transport. Our data shows that the IGF2BP family regulates the expression of crucial pluripotency factors in stem cells. Thus, it would be interesting to know if IGF2BP1 modulates the expression level of these pluripotency factors through regulation at the post-transcriptional level. Future work is needed to elucidate the coordination and differences between the role of the IGF2BP family in stem cells.

The m6A modification present on mRNA transcripts of pluripotency factors plays a crucial role in regulating pluripotency [13, 43, 44]. We observed that the m6A modification tends to have an enriched distribution in the coding CDS region as well as at translation termination sites of mRNAs, which is similar to the results reported by Chen et al. [45]. We also found that the 5’ and 3’ UTR regions of these four genes were not modified by m6A during stem cell reprogramming. Future studies will focus on the specific mechanism of m6A modification in stem cell reprogramming. Assays, like m6A-CLIP-seq (m6A-IP followed by NGS), would be needed to show that the changes in transcript levels of stem-cell factors are in fact regulated by the m6A marks through the effector IGF2BP1.

Dynamic, rapid alteration of gene expression ensures the flexibility of stem cell differentiation and reprogramming [46]. The m6A RNA modification was reported to help maintain the balance between lineage priming and pluripotency factors, thereby resulting in a rapid response to external cues during times of cell fate transition [47]. In our study, mRNAs of stem cell core pluripotency factors *SOX2*, *POU5F1* (*OCT4*), and *NANOG* all have the m6A modification. The typical m6A sequence motifs have also been identified after analysis of m6A enrichment regions in human umbilical cord mesenchymal stem cells (Table. S4). Consistent with our data, Chen et al. recently showed that *SOX2* exhibits a high degree of m6A methylation in hESCs [47]. They demonstrated temporal SOX2-specific m6A demethylation using dCas13a-catalytic domain of ALKBH5 (AlkB Homolog 5, RNA Demethylase, a demethylase or “eraser”) that inhibited nuclei‐to‐cytoplasm transport of *SOX2* mRNA, thereby controlling the differentiation of hESCs [47]. However, our results are in contrast to Batista’s report that revealed that OCT4 lacked the m6A modification while the mRNAs for *NANOG*, *SOX2*, and *KLF4* were modified with m6A in mESCs and hESCs [13]. However, future studies will be needed to learn if *IGF2BP1* is also involved in the determination of stem cell fate, specifically whether the downregulation and overexpression of *IGF2BP1* will affect reprogramming, using assays like alkaline phosphatase staining and iPSC colonies formation. In addition, this study is focused primarily on *OCT4* and *SOX2*. It is still not clear whether IGF2BPs may regulate expression of other stem genes, e.g., *MYC*, a well-known target of IGF2BP1 which interacts in an m6A dependent manner [48]. Future studies are needed to characterize the specificities and functional redundancies between IGF2BPs family. In this study, we knocked down the IGF2BPs separately. It would be interesting to examine if simultaneous knockdown of all three IGF2BPs would have an additive or detrimental effect on stem cell fate.

In summary, we identified *IGF2BP1* as an important pluripotency-associated m6A mRNA methylation effector in both human and mouse. IGF2BP1, an m6A reader, is involved in regulating the translational and expression levels of pluripotency factors, especially *SOX2*, which is required for proper reprogramming and stemness maintenance. These findings set the stage for dissecting the role of the m6A RNA modification in developmental fate transitions. Since *IGF2BP1* serves as an important regulator of pluripotency, it will also be interesting to expand this study by exploring its function in a knockout IGF2BP1 mouse model.

### Electronic supplementary material

Below is the link to the electronic supplementary material.

Supplementary Material 1

## Data Availability

Not applicable.
