# Supplementary material for: Profiling the role of m6A effectors in the regulation of pluripotent reprogramming
Source: Hum Genomics. 2024 Apr 2;18:33. doi: 10.1186/s40246-024-00597-6 (PMC10986062; doi:10.1186/s40246-024-00597-6)
Supplement: Supplementary file 1 — Supplementary Material 1 [file 40246_2024_597_MOESM1_ESM.docx]

### **Supplemental Figures**


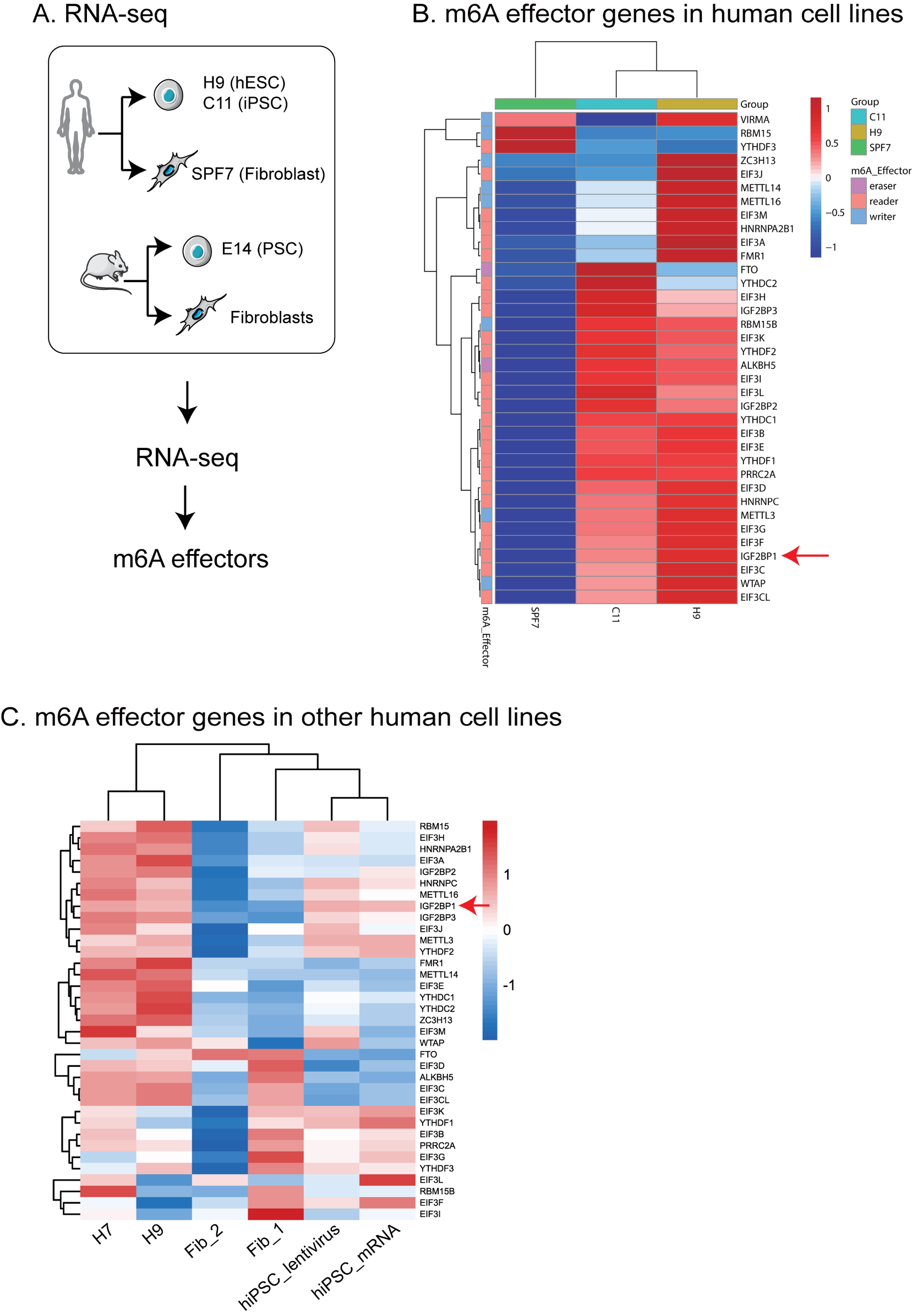


**Figure S1. Differential expression of m6A effector genes in pluripotent stem cell lines. A.** Schematic diagram of the RNA-seq using human and mouse stem cells and fibroblasts. hESC: human embryonic stem cell; iPSC: induced pluripotent stem cells; PSC: Pluripotent stem cells. **B.** Heatmap depicting expression level of m6A effector genes in human stem cells (H9 and C11) and fibroblast (SPF7). The expression levels were normalized by the row. **C.** Heatmap depicting expression level of m6A effector genes from six human samples [1], including two fibroblast cell lines, two ESC lines (H9 and H7), two hiPSCs. The expression levels were normalized by the row.

**
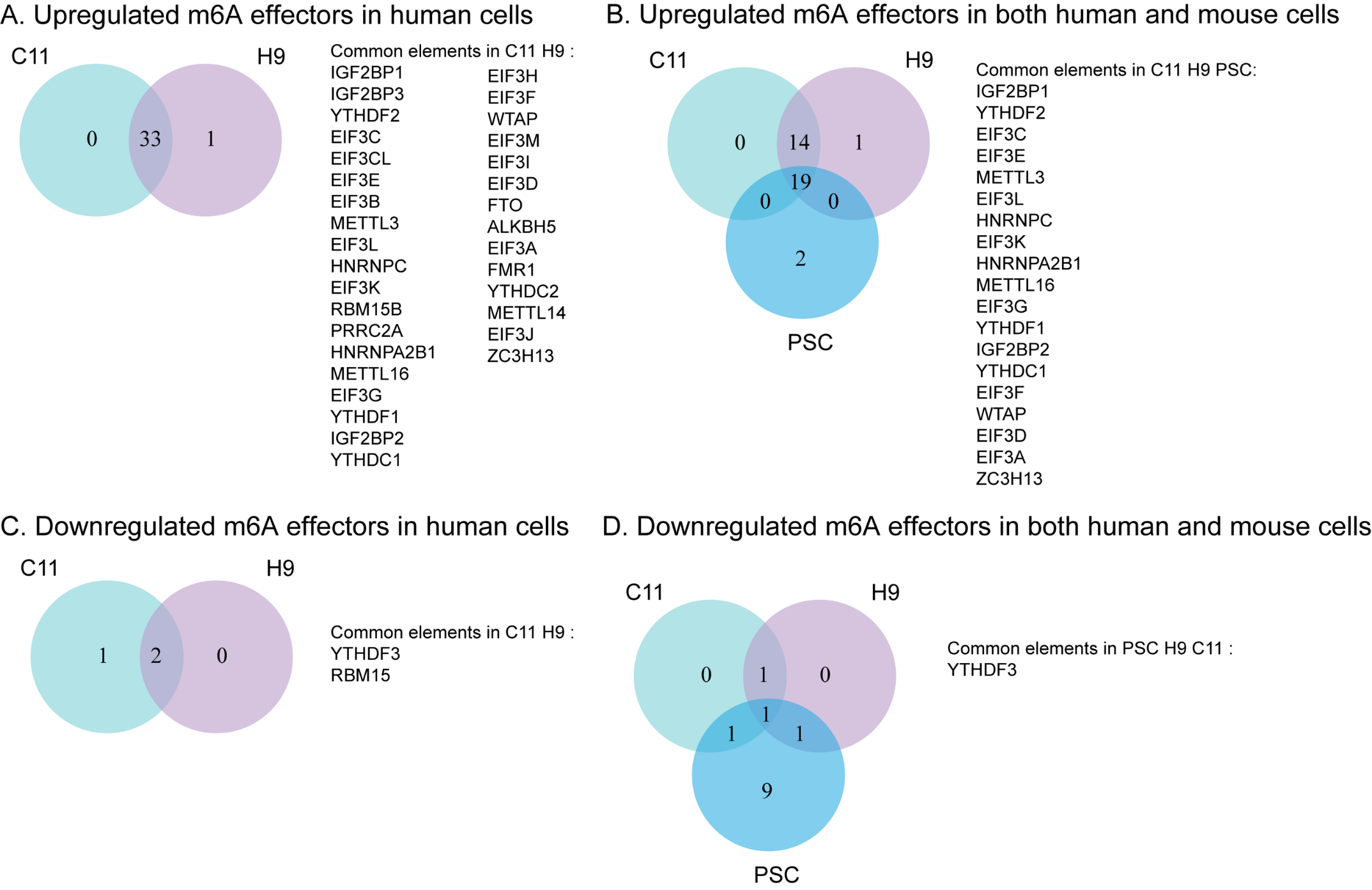
**

**Figure S2. Integrated analysis of m6A effectors in human and mouse cells.** A. Integration of upregulated m6A effectors in human cells H9 and C11. RNA-Seq was initially used to identify the upregulated m6A effectors (>2-fold, *P*<0.05) in stem cells. The upregulated m6A effectors were then integrated between two human stem cell lines using a VENN program. Such integration generated a list of 33 common upregulated m6A effectors. **B.** Integration of upregulated m6A effectors in both human (H9 and C11) and mouse (PSC) stem cells. The VENN program shows there are 19 common upregulated m6A effectors in both human and mouse stem cells. **C.** Integration of downregulated m6A effectors in human stem cells (H9 and C11). Y*THDF3* and *RBM15* are downregulated in both human and mouse stem cells. **D.** Integration of downregulated m6A effectors in both human (H9 and C11) and mouse (PSC) stem cells. *YTHDF3* is the only downregulated m6A effector in both human and mouse stem cells.

**
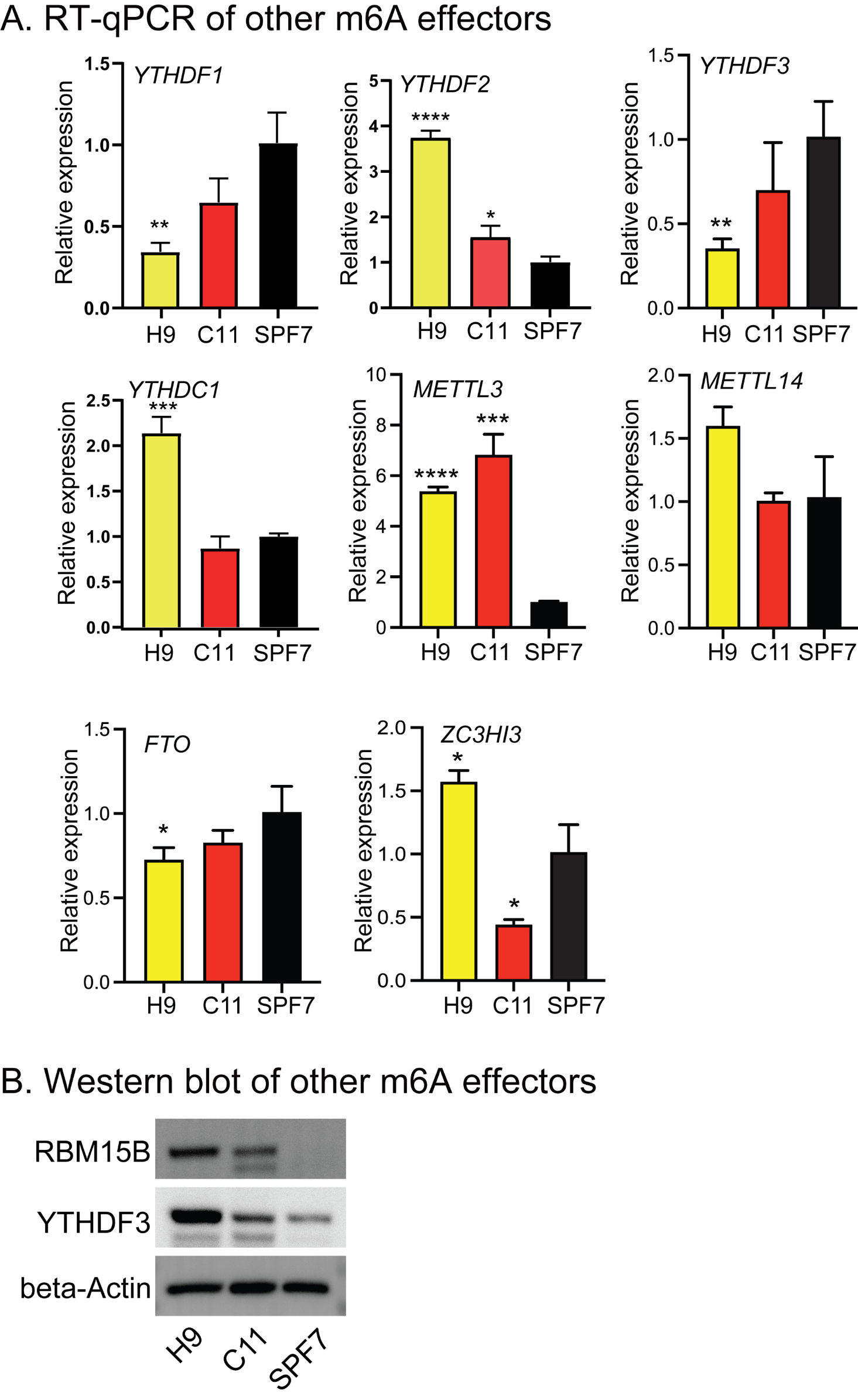
**

**Figure S3. Expression of other m6A effectors in pluripotent stem cell lines. A.** Human stem cell (H9 and C11) and fibroblasts (SPF7) were collected and then the expression of *YTHDF1-3*, *YTHDC1*, and *METTL3* was evaluated by real-time RT-PCR. The expression levels were normalized using β-Actin. **B.** Western blot results of other m6A effectors including RBM15B and YTHDF3.

**
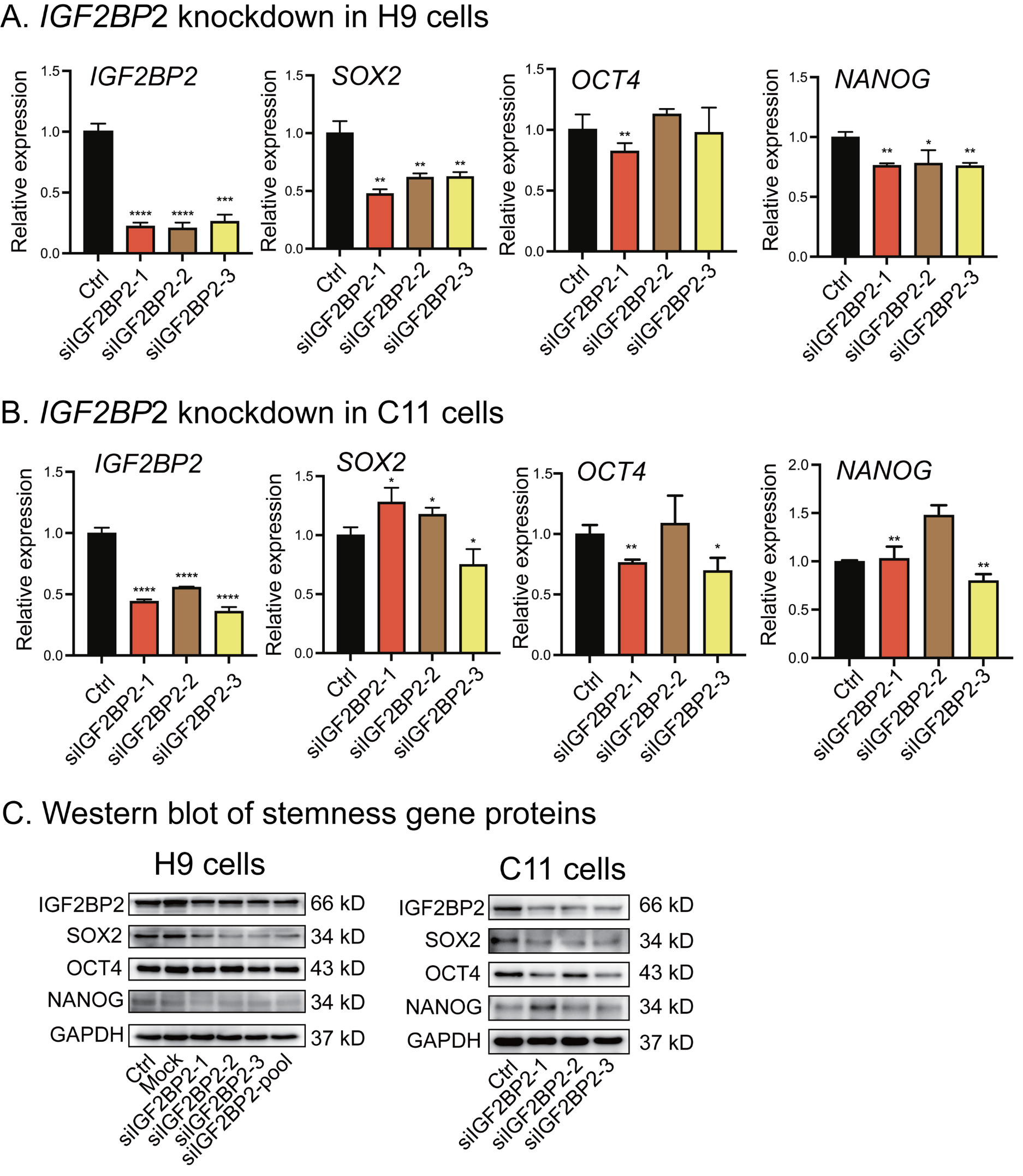
**

**Figure S4. The role of *IGF2BP2* in the regulation of stemness genes**. **A-B.** Quantitation of stemness genes by real-time PCR in H9 (A) and C11 (B) cells. After *IGF2BP2* siRNA transfection, cells were collected 48 hours for real-time PCR. β-Actin was used as the control. **C.** Western blot of stemness genes. Cells were collected for Western blotting 72 hours after siIGF2BP1 treatment in H9 cells


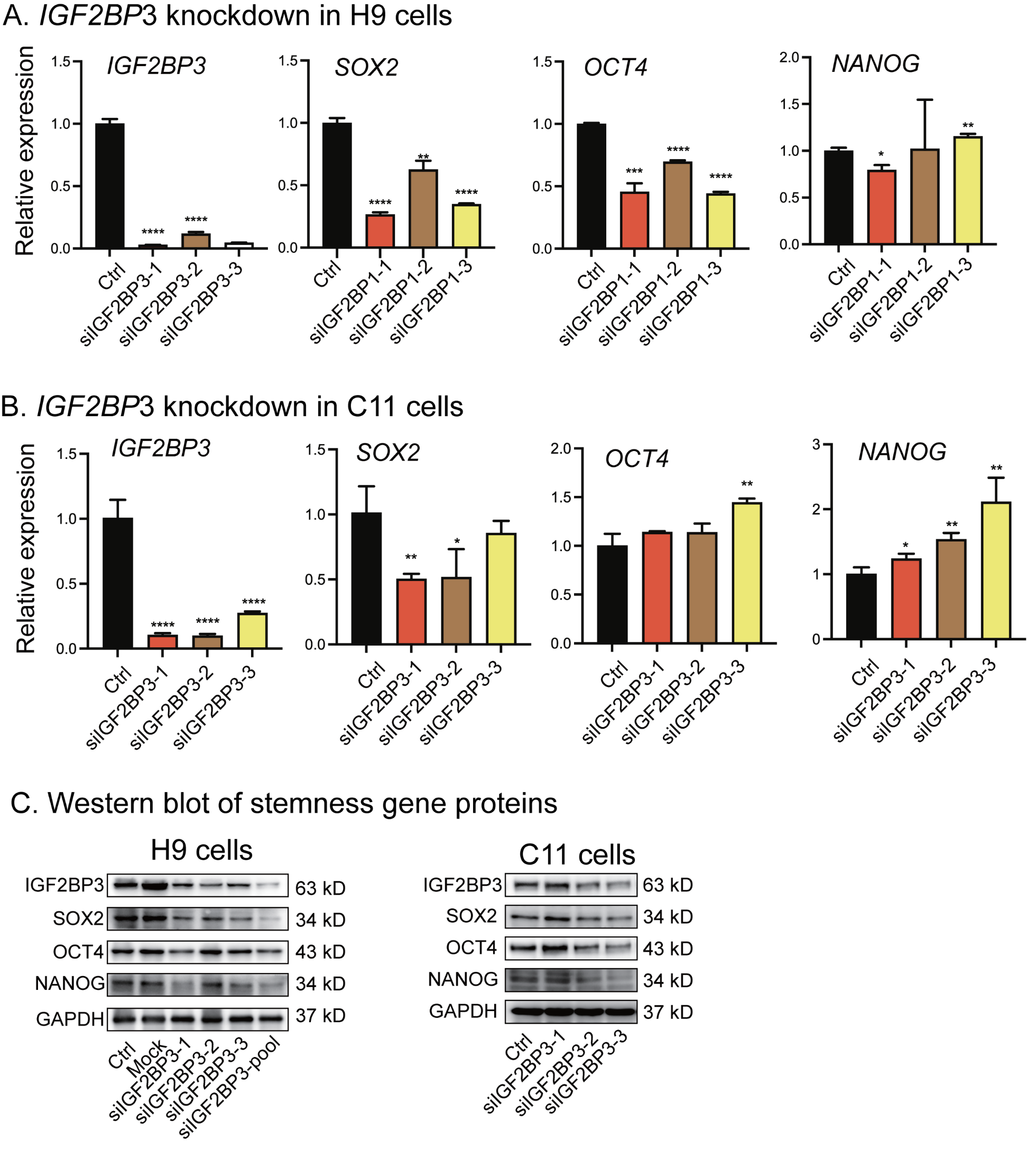


**Figure S5. The role of *IGF2BP3* in the regulation of stemness genes**. **A-B.** Quantitation of stemness genes by real-time PCR in H9 (A) and C11 (B) cells. After *IGF2BP3* siRNA transfection, cells were collected 48 hours for real-time PCR. β-Actin was used as the control. **C.** Western blot of stemness genes. Cells were collected for Western blotting 72 hours after siIGF2BP1 treatment in H9 cells.

**
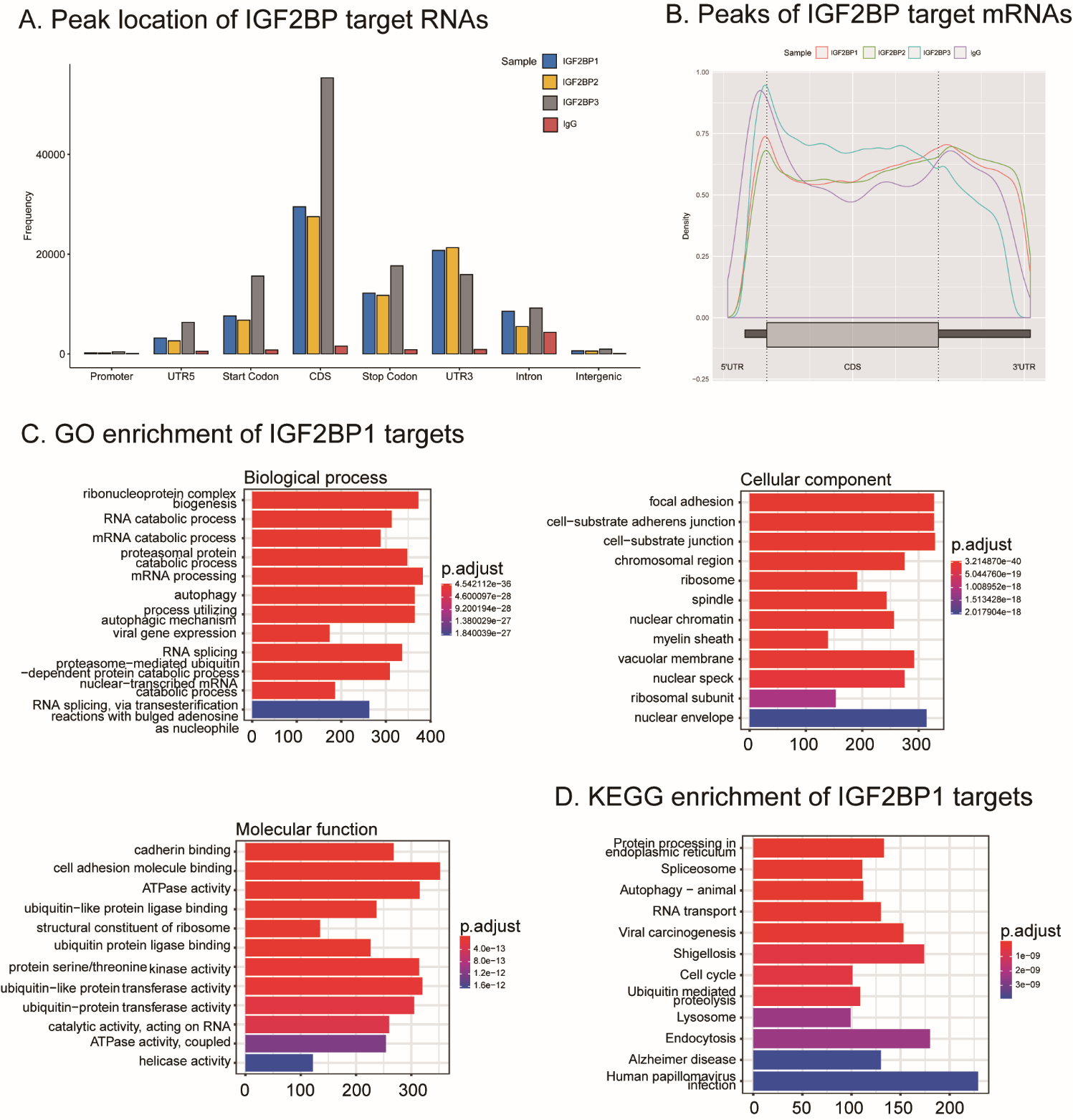
**

**Figure S6. CLIP identification of IGF2BP1 target genes** [2]**.** Gene ontology (GO, **A-B:** Peak location of IGF2BP1 target mRNAs. **C-D.** Gene ontology (GO, **C**) and Kyoto Encyclopedia of Genes and Genomes (KEGG, **D**) analyses of transcripts with significantly enriched IGF2BP1 binding.


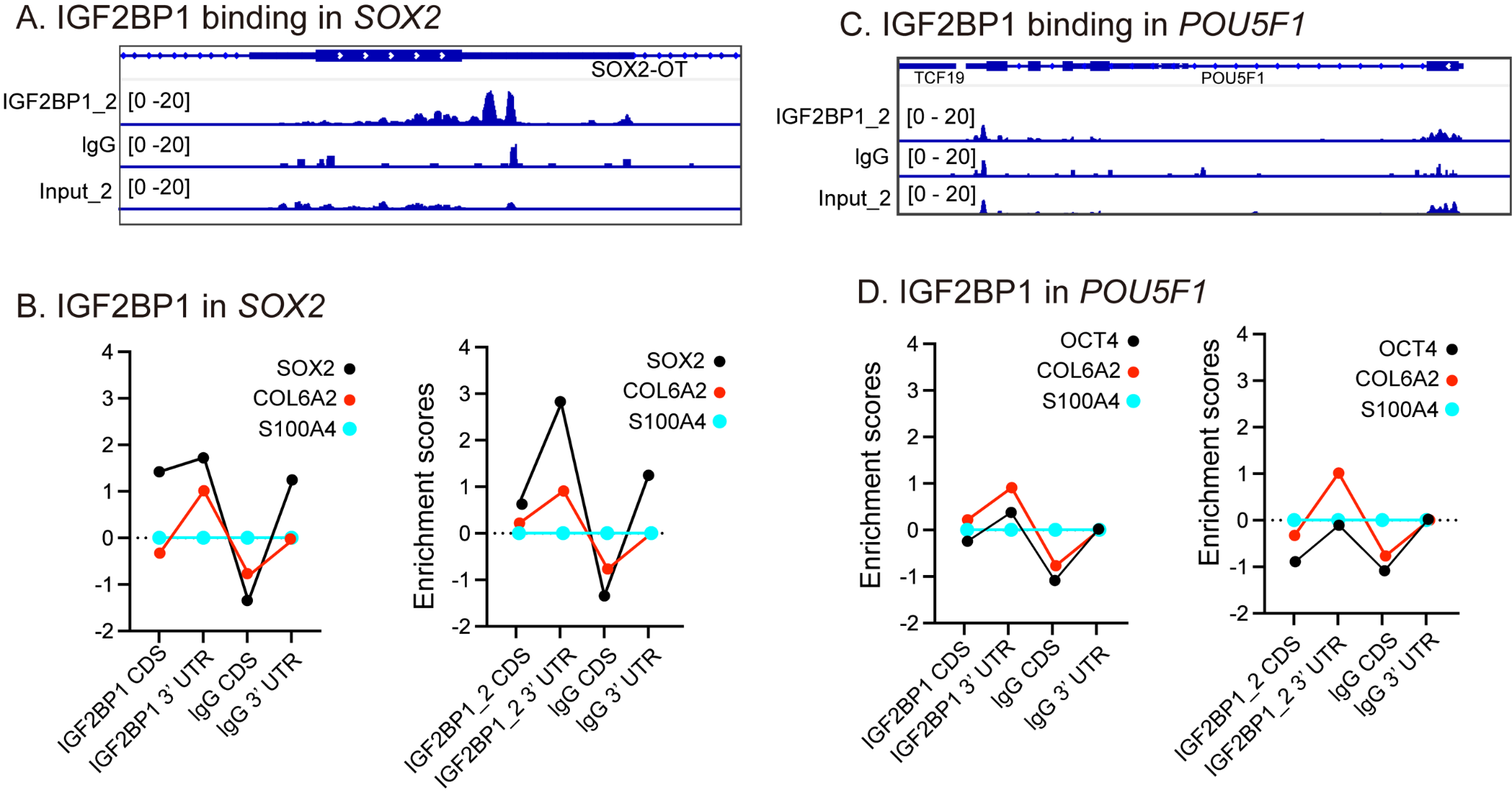


**Figure S7. IGF2BPs bind to stem genes in pluripotent stem cells by Technical duplication.** **A and C.** IGF2BP1-binding sites identified and eCLIP signal in H9 cell at the *SOX2* (**A**), and *POU5F1* (*OCT4*, **C**) locus. **B and D.** Fold-enrichment of IGF2BP1-binding sites in *SOX2* (**B**), *OCT4* (**D**) and two control genes (*COL6A2* and *S100A4*) in H9 cells.


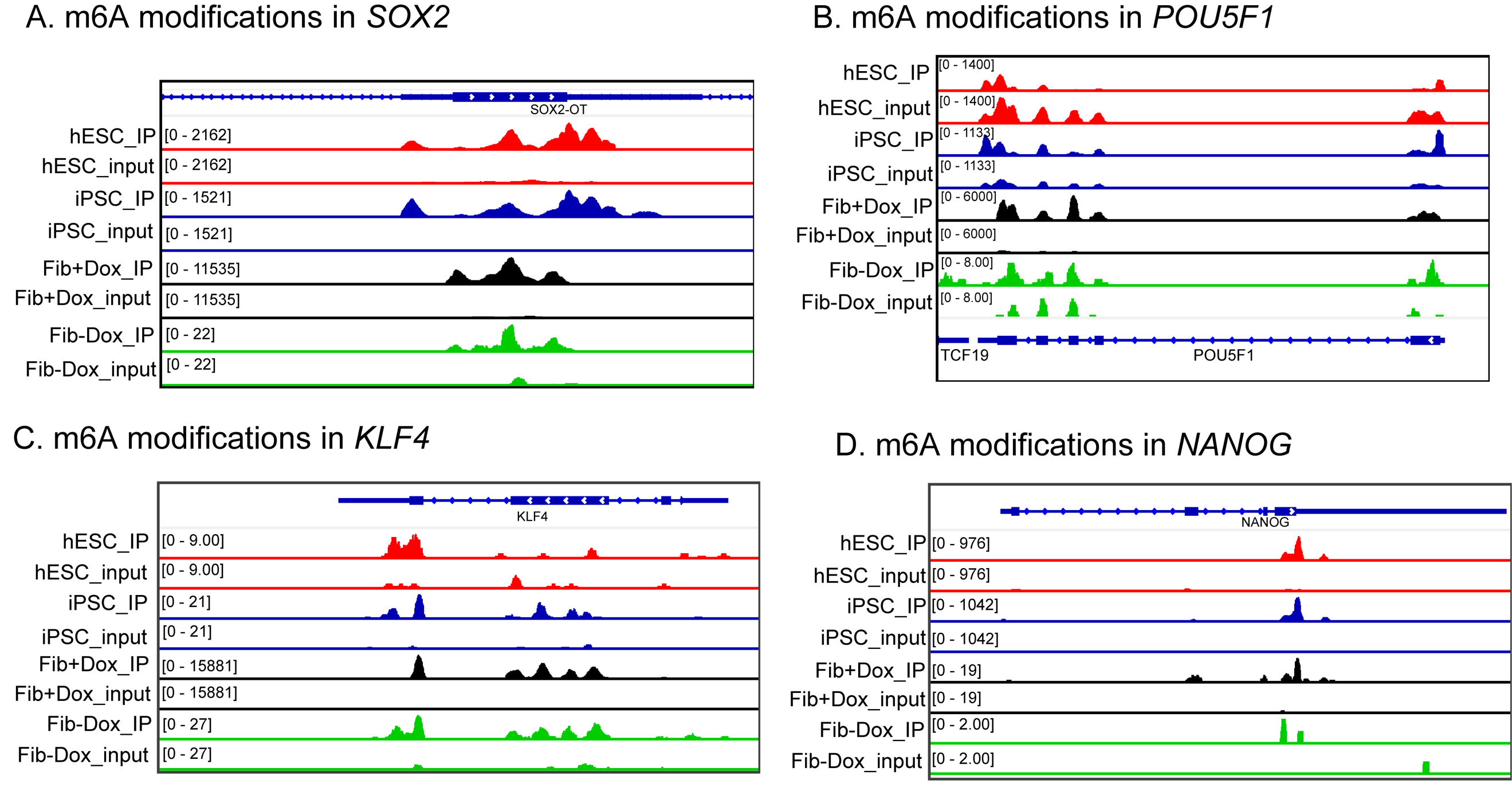


**Figure S8*.* Reprogramming-associated m6A modifications in four stem gene transcripts.** The m6A signal in stem gene transcripts involved in stem cell reprogramming. m6A-IP enrichment signals were the read depth of m6A immunoprecipitation data in human hESC (red), iPSC (fibroblasts fully reprogrammed into iPSC, blue), GSM1339403 fibro+Dox (5 days after fibroblasts post induction with Dox, black), GSM1339405 fibro+Dox (5 days after fibroblasts not inducing with Dox, green). Transcript regions are depicted as coding exons which are represented by blocks connected by horizontal lines representing introns.

### **Table S1. Oligonucleotide primers used for qPCR**

| ID | Oligo Name | Oligo sequence (5’ – 3’) |
| --- | --- | --- |
| *IGF2BP1* | IGF2BP1_F | CCTGCTGGCTCAGTATGGT |
|  | IGF2BP1_R | GACATTCACCACTGCCGTCTC |
| *IGF2BP2* | IGF2BP2_F | TGGCTTGGTTGGAAGACTGATTGG |
|  | IGF2BP2_R | GCCTGGGTTGGGATGAAGAGATTC |
| *IGF2BP3* | IGF2BP3_F | GCTCTATCAGTCGGTGCCATCATC |
|  | IGF2BP3_R | ACTTTAGCATCTGGTGCTTCCGC |
| *YTHDF1* | YTHDF1_F | GAGCCCTACCTTACTGGACAG |
|  | YTHDF1_R | GATGGTCTCCGTTACTGAGCTGTC |
| *YTHDF2* | YTHDF2_F | CACTCAGGAAGTGCCTCTGG |
|  | YTHDF2_R | CCACGACCTTGACGTTCCTTT |
| *YTHDF3* | YTHDF3_F | TGACAACAAACCGGTTACCA |
|  | YTHDF3_R | TGTTTCTATTTCTCTCCCTACGC |
| *YTHDC1* | YTHDC1_F | CTCAGGACATCATCCAGTACCACATG |
|  | YTHDC1_R | ACTTCTCCGGCCACTGACAAC |
| *METTL3* | METTL3_F | GAGGAGTGCATGAAAGCCAG |
|  | METTL3_R | GGCCTCAGAATCCATGCAAG |
| ***METTL14*** | METTL14_F | GACGGGGACTTCATTCATGC |
|  | METTL14_R | CCAGCCTGGTCGAATTGTAC |
| ***FTO*** | FTO_F | AGACACCTGGTTTGGCGATA |
|  | FTO_R | CCAAGGTTCCTGTTGAGCAC |
| ***ZC3H13*** | ZC3H13_F | CTTCAGACGGGATTCTGCAATTCG |
|  | ZC3H13_R | GAGCATGGCAAGTAGTCTTCCG |
| *SOX2* | hSOX2_F | ATGACCAGCTCGCAGACCTAC |
|  | hSOX2_R | TTGACCACCGAACCCATGGAG |
| *OCT4* | hOCT4_F | TCGAGAACCGAGTGAGAGG |
|  | hOCT4_R | GAACCACACTCGGACCACA |
| *NANOG* | NANOG_F | TTTGTGGGCCTGAAGAAAACT |
|  | NANOG_R | AGGGCTGTCCTGAATAAGCAG |
| *β-actin* | JH7132 hβ-Actin F2 | CACCCAGCACAATGAAGATCAAGAT |
|  | JH7133 hβ-Actin R2 | CCAGTTTTTAAATCCTGAGTCAAGC |

### Abbreviations: IGF2BP: insulin-like growth factor-2 mRNA-binding proteins; YTHDF1, YTH N6-methyladenosine RNA binding protein 1; YTHDF2, YTH N6-methyladenosine RNA binding protein 2; YTHDF3, YTH N6-methyladenosine RNA binding protein 3; YTHDC1, YTH domain containing 1; METTL3, methyltransferase 3, N6-adenosine-methyltransferase complex catalytic subunit; SOX2: SRY-box transcription factor 2; POU5F1/OCT4, POU class 5 homeobox 1; NANOG: Nanog homeobox.

**Table S2.** The expression of m6A effector genes from RNA-seq data of human cell lines.

| Gene name | C11_FPKM | H9_FPKM | SPF7_FPKM | H9/SPF7 Foldchange | C11/SPF7_Foldchange |
| --- | --- | --- | --- | --- | --- |
| IGF2BP1 | 123.619354 | 162.55249 | 1.00768 | 161.3136015 | 122.6771932 |
| IGF2BP3 | 26.686025 | 18.936229 | 3.0432635 | 6.222342889 | 8.768884127 |
| YTHDF2 | 31.5217445 | 27.716639 | 3.6159895 | 7.665021981 | 8.717321911 |
| EIF3C | 95.75174 | 127.087898 | 29.359098 | 4.328739868 | 3.261399243 |
| EIF3CL | 101.692513 | 131.080521 | 31.791262 | 4.123161924 | 3.198756721 |
| EIF3E | 75.7339455 | 79.273388 | 26.545343 | 2.986338809 | 2.853003086 |
| EIF3B | 67.0309395 | 70.326362 | 25.9313495 | 2.712020907 | 2.584938339 |
| METTL3 | 34.332409 | 39.464725 | 13.612632 | 2.899125239 | 2.522099253 |
| EIF3L | 151.791672 | 127.753181 | 60.320484 | 2.117907094 | 2.516419994 |
| HNRNPC | 123.264916 | 138.3599935 | 54.878384 | 2.521211148 | 2.246146971 |
| EIF3K | 136.942673 | 131.137741 | 61.091465 | 2.146580394 | 2.241600738 |
| RBM15B | 27.93227 | 26.815311 | 12.638895 | 2.121649954 | 2.21002469 |
| PRRC2A | 116.998291 | 115.279846 | 55.812321 | 2.065490987 | 2.096280694 |
| HNRNPA2B1 | 181.797745 | 283.276581 | 89.120506 | 3.178579136 | 2.039909255 |
| METTL16 | 3.271333 | 5.287748 | 1.651537 | 3.201713313 | 1.980780933 |
| EIF3G | 100.019463 | 112.76091 | 52.561611 | 2.145309245 | 1.902899494 |
| YTHDF1 | 32.42733 | 32.622555 | 17.098873 | 1.907877496 | 1.896460077 |
| IGF2BP2 | 49.277172 | 45.200245 | 26.545118 | 1.702770543 | 1.856355357 |
| YTHDC1 | 8.874834333 | 9.087051667 | 4.889197667 | 1.858597726 | 1.815192377 |
| EIF3H | 23.4557015 | 19.600379 | 13.1144135 | 1.494567714 | 1.788543689 |
| EIF3F | 28.737389 | 32.449062 | 17.56049 | 1.847844906 | 1.636479905 |
| WTAP | 28.208548 | 32.993774 | 17.384941 | 1.897836409 | 1.622585202 |
| EIF3M | 28.413267 | 39.377102 | 17.736378 | 2.220132092 | 1.601976852 |
| EIF3I | 104.619476 | 100.740929 | 68.456589 | 1.471603106 | 1.528260136 |
| EIF3D | 43.7414185 | 45.986752 | 30.1897845 | 1.523255391 | 1.448881442 |
| FTO | 5.155803333 | 4.022985667 | 3.607502667 | 1.115171918 | 1.429189057 |
| ALKBH5 | 45.124451 | 43.643513 | 32.42976 | 1.345785877 | 1.391451895 |
| EIF3A | 54.623466 | 79.727242 | 44.565449 | 1.78899223 | 1.225690916 |
| FMR1 | 11.547412 | 15.157701 | 9.668626 | 1.567720274 | 1.194317786 |
| YTHDC2 | 10.660805 | 10.000368 | 9.570997 | 1.04486168 | 1.113865671 |
| METTL14 | 4.157364 | 4.670544 | 3.750522 | 1.245305054 | 1.1084761 |
| EIF3J | 10.379283 | 15.470542 | 9.773077 | 1.582975556 | 1.062028162 |
| ZC3H13 | 11.940234 | 22.425119 | 11.640199 | 1.926523679 | 1.025775762 |
| VIRMA | 4.078496 | 5.140094333 | 4.927753667 | 1.043090763 | 0.827658255 |
| YTHDF3 | 8.981175 | 7.949505 | 18.248938 | 0.435614664 | 0.492147817 |
| RBM15 | 1.688759 | 2.011856 | 9.592432 | 0.209733673 | 0.176051183 |

**Table S3**. **Oligonucleotide primers used for RIP and CLIP**

| ID | Primers | Oligo sequence (5’ – 3’) |
| --- | --- | --- |
| *POU5F1* | Intron 2-F | AGGAGTATCCCTGAACCTAG |
|  | Intron 2-R | GTGACCCTGAGATCCAAGCT |
|  | Exon 1-F | CTTGGAGACCTCTCAGCCTGA |
|  | Exon 1-R | TTCTCCTTCTCCAGCTTCACGG |
|  | Exon 5-F1 | CGAGTGTGGTTCTGTAACCGG |
|  | Exon 5-R1 | AAAGGACACTGGTCCCCCTGA |
|  | Exon 5-F2 | AGCCCTCACTTCACTGCACTGT |
|  | Exon 5-R2 | CTAGAAGGGCAGGCACCTCAGT |
| *SOX2* | CDS-F1 | ACAACATGATGGAGACGGAGCT |
|  | CDS-R1 | CCACACCATGAAGGCATTCATG |
|  | CDS-F2 | AGAAGGATAAGTACACGCTGCC |
|  | CDS-R2 | CATCATGCTGTAGCTGCCGTTG |
|  | CDS-F3 | ATGACCAGCTCGCAGACCTAC |
|  | CDS-R3 | TTGACCACCGAACCCATGGAG |
|  | CDS-F4 | GGGACATGATCAGCATGTATCT |
|  | CDS-R4 | CAGTGTGCCGTTAATGGCCGTG |
|  | 3’ UTR-F1 | ATCCCATCACCCACAGCAAATG |
|  | 3’UTR-R1 | AGAAGTCCAGGATCTCTCTCAT |
|  | 3’UTR-F2 | CGAACCATCTCTGTGGTCTTGT |
|  | 3’UTR-R2 | ATTACCAACGGTGTCAACCTGC |

1. Churko JM, Lee J, Ameen M, Gu M, Venkatasubramanian M, Diecke S, et al. Transcriptomic and epigenomic differences in human induced pluripotent stem cells generated from six reprogramming methods. Nat Biomed Eng. 2017;1(10):826-37; doi: 10.1038/s41551-017-0141-6.

2. Conway AE, Van Nostrand EL, Pratt GA, Aigner S, Wilbert ML, Sundararaman B, et al. Enhanced CLIP Uncovers IMP Protein-RNA Targets in Human Pluripotent Stem Cells Important for Cell Adhesion and Survival. Cell Rep. 2016;15(3):666-79; doi: 10.1016/j.celrep.2016.03.052.

**Table S4. Enriched motif in** **human umbilical cord mesenchymal stem cells.** The methylated RNA immunoprecipitation sequencing (MeRIP-seq) of three human umbilical cord mesenchymal stem cell samples (GSM5224627, GSM5224629, GSM5224631) has been collected. RNAmod was used for de novo discovery of the methylation motif of mRNA.

| Motif | Enrichment P-value |
| --- | --- |
| 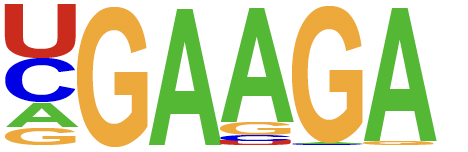 | 1e-358 |
| 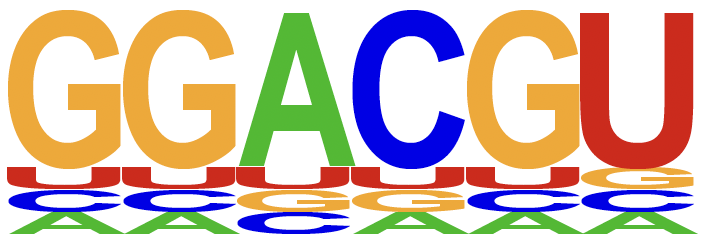 | 1e-331 |
| 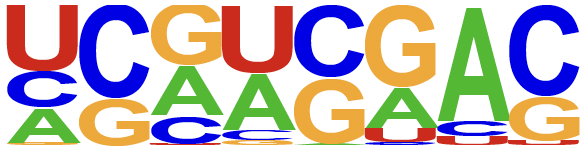 | 1e-357 |
| 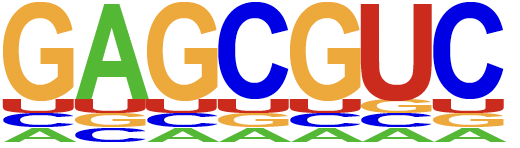 | 1e-344 |
| 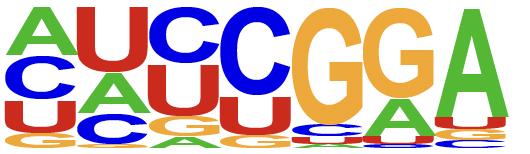 | 1e-342 |
| 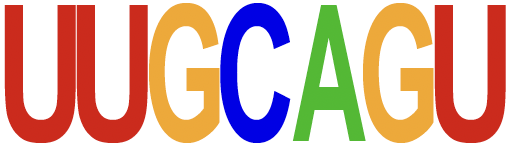 | 1e-340 |
| 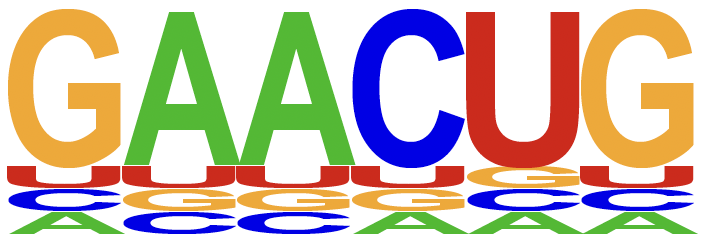 | 1e-311 |
| 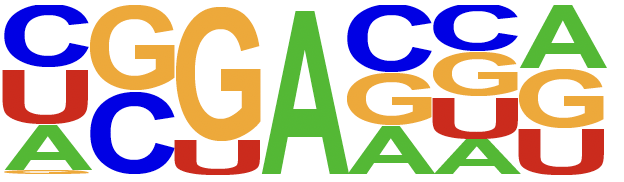 | 1e-416 |
| 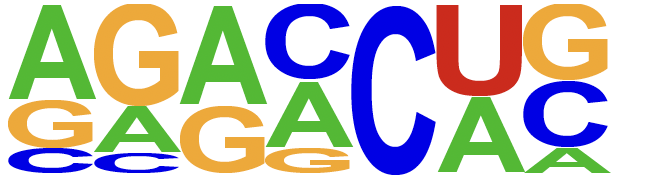 | 1e-248 |
| 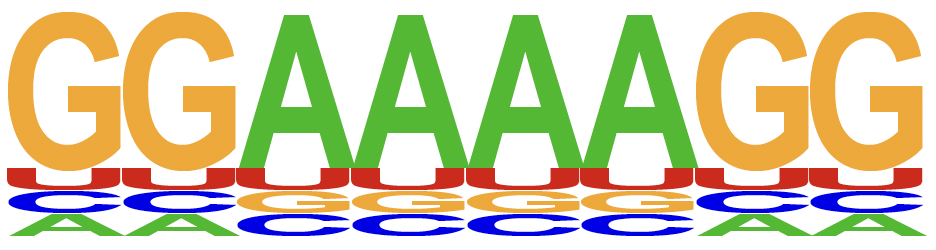 | 1e-328 |
